# Expression of Circ_Satb1 Is Decreased in Mesial Temporal Lobe Epilepsy and Regulates Dendritic Spine Morphology

**DOI:** 10.3389/fnmol.2022.832133

**Published:** 2022-03-03

**Authors:** Andreia Gomes-Duarte, Morten T. Venø, Marina de Wit, Ketharini Senthilkumar, Mark H. Broekhoven, Joëlle van den Herik, Fleur R. Heeres, Daniëlle van Rossum, Mateja Rybiczka-Tesulov, Ivano Legnini, Peter C. van Rijen, Pieter van Eijsden, Peter H. Gosselaar, Nikolaus Rajewsky, Jørgen Kjems, Vamshidhar R. Vangoor, R. Jeroen Pasterkamp

**Affiliations:** ^1^Affiliated Partner of the European Reference Network EpiCARE, Department of Translational Neuroscience, UMC Utrecht Brain Center, University Medical Center Utrecht, Utrecht University, Utrecht, Netherlands; ^2^Interdisciplinary Nanoscience Center, Department of Molecular Biology and Genetics, Aarhus University, Aarhus, Denmark; ^3^Omiics ApS, Aarhus, Denmark; ^4^Systems Biology of Gene Regulatory Elements, Berlin Institute for Medical Systems Biology, Max Delbrück Center for Molecular Medicine in the Helmholtz Association, Berlin, Germany; ^5^Department of Neurology and Neurosurgery, UMC Utrecht Brain Center, University Medical Center Utrecht, Utrecht University, Utrecht, Netherlands

**Keywords:** RNA-sequencing, circular RNA, dendritic spine, hippocampus, epilepsy

## Abstract

Mesial temporal lobe epilepsy (mTLE) is a chronic disease characterized by recurrent seizures that originate in the temporal lobes of the brain. Anti-epileptic drugs (AEDs) are the standard treatment for managing seizures in mTLE patients, but are frequently ineffective. Resective surgery is an option for some patients, but does not guarantee a postoperative seizure-free period. Therefore, further insight is needed into the pathogenesis of mTLE to enable the design of new therapeutic strategies. Circular RNAs (circRNAs) have been identified as important regulators of neuronal function and have been implicated in epilepsy. However, the mechanisms through which circRNAs contribute to epileptogenesis remain unknown. Here, we determine the circRNA transcriptome of the hippocampus and cortex of mTLE patients by using RNA-seq. We report 333 differentially expressed (DE) circRNAs between healthy individuals and mTLE patients, of which 23 circRNAs displayed significant adjusted *p*-values following multiple testing correction. Interestingly, hippocampal expression of circ_Satb1, a circRNA derived from special AT-rich sequence binding protein 1 (*SATB1*), is decreased in both mTLE patients and in experimental epilepsy. Our work shows that circ_Satb1 displays dynamic patterns of neuronal expression *in vitro* and *in vivo*. Further, circ_Satb1-specific knockdown using CRISPR/CasRx approaches in hippocampal cultures leads to defects in dendritic spine morphology, a cellular hallmark of mTLE. Overall, our results identify a novel epilepsy-associated circRNA with disease-specific expression and previously unidentified cellular effects that are relevant for epileptogenesis.

## Introduction

Epilepsy is a chronic neurological disease estimated to affect 70 million individuals worldwide (Singh and Trevick, 2016). Mesial temporal lobe epilepsy (mTLE), the most common form of focal epilepsy, involves the hippocampus, parahippocampal gyrus and amygdala structures of the temporal lobe (Noulhiane et al., 2006). MTLE is characterized by hippocampal dysfunction due to impairments in neural excitability and unprovoked temporal lobe seizures (Dalby and Mody, 2001). It has been associated with several underlying conditions including long-term epilepsy associated tumors (LEAT), cortical malformations, infections, febrile seizures, traumatic brain injury and genetic predisposition (Harvey et al., 1995; Berkovic et al., 1996; Englander et al., 2003; Duncan et al., 2006; Al Sufiani and Ang, 2012; Barkovich et al., 2012; Thom et al., 2012). To date, anti-epileptic drugs (AEDs) persist as the primary treatment against seizures and mTLE (Shinnar and Berg, 1996). Unfortunately, due to the progressive nature of mTLE, seizures initially controlled by AEDs can become untreatable over time (French et al., 1993; Camfield and Camfield, 1996; Devinsky et al., 2018). This condition, also known as refractory epilepsy, is estimated to affect 70–80% of mTLE patients (Benbadis and Semah, 1999; Kurita et al., 2016). In these cases, surgical removal of the epileptogenic region can help with seizure control (Miller and Hakimian, 2013). However, resective surgery does not guarantee a postoperative seizure-free condition (Engel, 1996) and can cause neurologic complications, such as impairment of visual, motor, speech, and memory tasks (Hader et al., 2013). Furthermore, refractory patients experience a severe condition in which the occurrence of disabling seizures occurs along with other comorbidities such as depression, anxiety and cognitive deterioration (Laxer et al., 2014). Ultimately, this results in decreased quality of life and increased risk of premature death (Laxer et al., 2014). Therefore, there is an urgent need for improved insight into the pathogenesis of mTLE to develop new treatments for this disabling disease.

A growing body of experimental evidence implicates non-coding RNAs (ncRNAs) in epilepsy. NcRNAs are deregulated in both human and experimental epilepsy, and have been linked to disease-associated changes in processes such as inflammatory and immune responses, apoptosis, neurogenesis, and neural plasticity (Henshall and Kobow, 2015; Henshall et al., 2016; Shao and Chen, 2017; Villa et al., 2019; Gray et al., 2020). Circular RNAs (circRNAs) are a class of long ncRNAs that form a closed-loop structure, in which the 5′ and 3′ splice sites of a pre-mRNA are covalently bound (Jeck et al., 2013; Memczak et al., 2013). The most investigated mechanism-of-action of circRNAs is sponging of microRNAs (miRNAs) and RNA-binding proteins (RBPs) via sequence-specific interactions (Kristensen et al., 2019). CircRNAs are conserved across species and highly enriched in the mammalian brain (Rybak-Wolf et al., 2014). Furthermore, they are tightly regulated during neuronal differentiation and development, and display a specific subcellular distribution (e.g., in synapse) (Salzman et al., 2012; Jeck et al., 2013; Memczak et al., 2013; Rybak-Wolf et al., 2014; Venø et al., 2015; You et al., 2015). Unfortunately, their specific neuronal functions remain poorly understood. Our own work and that of others shows deregulated expression of circRNAs in mTLE patients and in experimental models of epilepsy (e.g., Gong et al., 2018; Lee et al., 2018; Li et al., 2018; Gray et al., 2020; Gomes-Duarte et al., 2021). For example, we have recently reported enrichment of circRNA deregulation at the transition to recurrent spontaneous seizures (Gomes-Duarte et al., 2021). Despite this recent progress, how circRNA deregulation contributes to the process of epileptogenesis remains largely unknown.

In the present study, we investigate changes in the expression of circRNAs in mTLE patients and experimental epilepsy, and study one of the deregulated circRNAs, circ_Satb1, in more detail. For example, we study circ_Satb1 expression during neural development and mimic the mTLE-associated downregulation of circ_Satb1 in hippocampal cultures by using CRISPR/CasRx technology. This unveils a role for this circRNA in the regulation of dendritic spine morphology. This is intriguing as changes in dendritic spines are an established hallmark of epilepsy (Swann et al., 2000; Wong, 2005; Wong and Guo, 2013). Together, our results provide a functional characterization of a mTLE-associated circRNA and for the first time implicate circRNAs in the regulation of dendritic spine morphology.

## Materials and Methods

### Patient Selection and Tissue Collection

Patient selection and hippocampal tissue collection from both patients and healthy donors was performed as previously reported (Kan et al., 2012; Vangoor et al., 2019). All patients and control donors provided a written informed consent for the use of their material and clinical information for research purposes (Vangoor et al., 2021). All procedures performed and the use of tissue and clinical information for research purposes were approved by the Institutional Review board of University Medical Center Utrecht. Postmortem tissue was obtained from the Netherlands Brain Bank. Use of postmortem tissue for research purposes was approved by the medical ethics board of the Amsterdam University Medical Center. During the course of the studies performed, group individuals were matched by sex and as much as possible by age, whenever the limiting age of autopsy controls allowed it. A summary of the clinical information from all patients and controls included in the study can be found in Table 1.

**TABLE 1:** Details of control individuals and mTLE patients used in this study.

Number	Gender	Age	Pathology	Anti-epileptic drugs (AEDs)	Studies
C1	M	48	Non-demented control	NA	RT-qPCR
C2	F	78	Non-demented control	NA	RT-qPCR
C3	M	93	Non-demented control	NA	RT-qPCR
C4	F	82	Non-demented control	NA	RT-qPCR
C5	F	72	Non-demented control	NA	RT-qPCR
C6	F	89	Non-demented control	NA	RT-qPCR
C7	F	75	Non-demented control	NA	RT-qPCR
E1	F	23	mTLE non-HS (no-HS)	PHT, CZP	RT-qPCR
E2	F	47	mTLE non-HS (no-HS)	LTG, OXC	RT-qPCR
E3	M	60	mTLE non-HS (no-HS)	PGB, OXC, HCT	RT-qPCR
E4	F	50	mTLE non-HS (no-HS)	GBP, CLO	RT-qPCR
E5	F	63	mTLE non-HS (no-HS)	LEV, CZP	RT-qPCR
E6	F	38	mTLE non-HS (no-HS)	CBZ, LEV	RT-qPCR
E7	M	44	mTLE non-HS (no-HS)	CBZ, BRV	RT-qPCR
E8	F	52	mTLE + HS (ILAE type 1)	CBZ, CZP, DZP	RT-qPCR
E9	M	41	mTLE + HS (ILAE type 1)	PHT, CZP, CBZ, LTG	RT-qPCR
E10	M	41	mTLE + HS (ILAE type 1)	CBZ	RT-qPCR
E11	M	42	mTLE + HS (ILAE type 1)	LEV, LTG	RT-qPCR
E12	F	49	mTLE + HS (ILAE type 1)	OXC, CLO, SER	RT-qPCR
E13	F	42	mTLE + HS (ILAE type 1)	LEV, LTG, PBT	RT-qPCR
C8	M	71	Non-demented control	NA	Western Blot
C9	M	62	Non-demented control	NA	Western Blot
C10	M	48	Non-demented control	NA	Western Blot
C11	M	70	Non-demented control	NA	Western Blot
C12	M	74	Non-demented control	NA	Western Blot
C13	F	50	Non-demented control	NA	Western Blot
E14	F	34	mTLE non-HS (no-HS)	CBZ	Western Blot
E15	F	40	mTLE non-HS (no-HS)	LEV, LTG, CBZ	Western Blot
E16	F	43	mTLE non-HS (no-HS)	PHE, LTG	Western Blot
E17	M	45	mTLE non-HS (no-HS)	PHE, LTG	Western Blot
E18	F	46	mTLE non-HS (no-HS)	CBZ, VPA	Western Blot
E19	M	46	mTLE non-HS (no-HS)	CBZ, VPA, TPR, FRS	Western Blot
C14	M	55	Non-demented control	NA	RNA-seq
C15	M	51	Non-demented control	NA	RNA-seq
C16	F	64	Non-demented control	NA	RNA-seq
C17	M	62	Non-demented control	NA	RNA-seq
C18	F	73	Non-demented control	NA	RNA-seq
E20	M	30	mTLE non-HS (no-HS)	CBZ, LTG, CLO	RNA-seq
E21	M	45	mTLE non-HS (no-HS)	CBZ, VPA, TPR	RNA-seq
E22	F	31	mTLE non-HS (no-HS)	CBZ, CLO	RNA-seq
E23	M	46	mTLE non-HS (no-HS)	LEV	RNA-seq
E24	F	42	mTLE non-HS (no-HS)	ZON, CBZ	RNA-seq

*M, male; F, female; HS, hippocampal sclerosis; NA, non-applicable. PHT, Phenytoin; CZP, Clonazepam; LTG, Lamotrigine; OXC, oxcarbazepine; PGB, Pregabalin; HCT, Hydrochlorothiazide; GBP, Gabapentin; CLO, Clobazam; LEV, Levetiracetam; CBZ, Carbamazepine; BRV, Brivaracetam; DZP, Diazepam; SER, Seroquel; PBT, Phenobarbital; PHE, Phenytoin; VPA, Valproic acid; TPR, Topiramate; FRS, Furosemide; ZON, Zonisamide.*

### Animal Experiments

All animal experiments were approved by local authorities in Utrecht (Animal Ethics Committee of Utrecht University) in compliance with Dutch law (Wet op de Dierproeven, 1996; revised 2014). All procedures were performed in accordance with EU regulations (Guideline 86/609/EEC; Directive 2010/63/EU).

#### Intracortical Kainic Acid Mouse Model

Intracortical kainic acid (KA) injections were performed as described previously (Bedner et al., 2015). Three months old (P90) C57BL/6 (JAX™) male mice were used for KA injections. Mice were injected with Caprofen (5 mg/kg, subcutaneous [s.c.]) 30 min before surgery. During surgery, mice were on gas anesthesia (isofluorane; 5% mixed with O2 [1.5–3%]) and their eyes were protected with eye ointment. Once mice were unconscious, body hair was removed from the skull and on the neck and the shaved areas were scrubbed with Sterilium (Hartmann). Mice were then fixed firmly on a dual arm stereotactic frame, containing a high-speed drill on one arm and a manual microinjection unit on the second arm. Stereotactic coordinates (Anterior/posterior: 2.0 mm; Medial/Lateral: 1.5 mm; Dorsal/Ventral: 1.7 mm) were marked using Bregma as a reference. A hole (approximately 0.7 mm) was drilled in the skull without damaging the dura and 70 nl of 20 mM KA (Tocris, United Kingdom) dissolved in 0.9% sterile NaCl (saline, control), was injected into the neocortex dorsal of the right dorsal hippocampus using a 0.5 μl microsyringe (Hamilton, Switzerland). Control mice were injected with 70 nl saline. Injections were performed over a period of 1 min using the micrometer screw, following which the syringe was left at the injection site for an additional 5 min to limit reflux along the needle track. Finally, the scalp incision was sutured (Ethicon, V926) and animals were transferred to a clean cage and kept on a heating plate until recovered from anesthesia. Painkiller (Caprofen [5 mg/kg, s.c.]) was administered after 12 hours (h) and until 2 days (d) after surgery. Similar as described by Bedner et al. (2015), we observed seizures up to 12 h after KA injection (termed as *status epilepticus* [SE]), with seizure events that exceeded 20 s, followed by a latent period of up to 14 days after injection. Then, the chronic stage started with the occurrence of convulsive seizures, termed tonic-clonic seizures (Racine scale III-V; Racine, 1972). Seizures were also assessed in an independent set of animals by performing electroencephalograph (EEG) recordings, as described previously (Bedner et al., 2015).

Kainic acid-injected and control mouse brains were collected after cold PBS perfusion at the following time points: 24 hours (h), 3, 5, 14, 30, and 90 days (d). For (immuno)stainings, 20 μm-thick cryosections of hippocampal tissue were prepared. Fresh-frozen slides were dried for 2 h at RT, fixed for 5 min (2× Xylene, 2 × 100% EtOH, 95% EtOH and 70% EtOH), stained with 0.1% Cresyl Fast Violet for 10 min, rinsed in water and dehydrated through a sequential series of EtOH (70, 96, and 100%). Finally, slides were dried for 5 min at RT and sections mounted using Entellan medium (Merck Millipore). For RNA extraction, hippocampus was dissected, snap frozen in liquid nitrogen and stored at −80°C until use.

### Cell Lines

#### Maintenance

Mouse neuroblastoma cell line (Neuro-2a, ATCC) was grown in Dulbecco’s modified Eagle’s medium (DMEM) containing high glucose (Thermo Fisher Scientific), supplemented with penicillin/streptomycin and 10% fetal calf serum. Cells were kept in a 5% CO_2_ atmosphere incubator at 37°C.

#### Transfection

Cells were transfected at early post-defrosting passages (P5-P15) when reaching 30–50% confluency. Assays were performed in a 24-well plate system (Corning). A total of 0.5 μg of plasmids and 2 μl of Lipofectamine 2000 were used at a final transfection volume of 100 μl. Gibco™ Opti-MEM™ (Thermo Fisher Scientific) was used as transfection medium. RNA or protein were isolated at 48 h following transfection.

### Culturing of Mouse Hippocampal Neurons

C57BL/6 mouse pups were used for culturing primary hippocampal neurons (PHN), as described previously (Van Battum et al., 2018; Vangoor et al., 2019). Briefly, C57BL/6 pups (P0) were decapitated and their brains collected in ice-cold L-dissection medium (Leibovitz’s L-15 supplemented with 7 mM HEPES; Thermo Fisher Scientific). The isolated hippocampi were subsequently trypsinized (0.25% trypsin in L15-HEPES medium) for 20 min at 37°C and dissociated into individual cells using fire-polished Pasteur pipettes and a 70 μm nylon cell strainer (Falcon^®^, Corning). Dissociated neurons (150–200 K/well) were then cultured in Neurobasal medium supplemented with B-27 (Thermo Fisher Scientific), L-glutamine, penicillin/streptomycin and β-mercaptoethanol and plated on poly-D-lysin- (20 μg/ml) and laminin- (40 μg/ml) coated coverslips in 12 well plates. PHN were kept in a 5% CO_2_ incubator at 37°C. Half of the culture medium was refreshed every 3–4 days and every procedure was performed on a heating plate.

For assessing RNA expression levels during *in vitro* differentiation, four coverslips containing PHN were lysed in 700 μl of QIAzol lysis reagent (Qiagen) at multiple days *in vitro* (DIV) (DIV7, 14 and 21). Collected neurons were stored at −80°C until RNA extraction.

### Activity Assays Using Mouse Hippocampal Neurons

#### KCl-Induced Depolarization

Primary hippocampal neurons were treated at DIV14 with either 20 mM KCl or NaCl at 37°C, by replacing half of the culture media. Following 4 h in KCl or NaCl conditions, PHN were transferred into a new plate containing half pre-depolarization medium and half fresh culture growth medium (supplemented Neurobasal medium). Four coverslips of PHN were lysed in 700 μl of QIAzol at 4 or 24 h post-treatment.

#### Mg^2+^ Depletion

Mg^2+^ depletion assays were performed as described previously (Hogg et al., 2019). PHN were treated with Mg^2+^- or depleted Mg^2+^-medium at DIV15. PHN media was replaced completely by 1 ml of Mg^2+^- or Mg^2+^-depleted medium and the plate was kept for 2 h at 37°C. Six coverslips of PHN were lysed in 700 μl of QIAzol at 2 h post-treatment. Samples were snap frozen at −80°C until RNA extraction.

#### Dendritic Spine Assays

DIV14 PHN were co-transfected with 0.5 μg of pU6_gRNA_circ_Satb1/pU6_gRNA_negative control (nc) and 0.5 μg of pCasRx_EGFP (Addgene #109049). Both DNA and Lipofectamine 2000 mixes were diluted in Neurobasal medium (Thermo Fisher Scientific) and incubated for 30 min at RT. In the meantime, the DIV14 neurons were placed in transfection media (Neurobasal medium with L-glutamine [Thermo Fisher Scientific, 1:400] and B-27™ Supplement [Thermo Fisher Scientific, 1:250]). At end of the 30 min, 200 μl DNA-lipofectamine complexes were added at multiple spots on the coverslips and incubated for 45 min at 37°C, after which the coverslips were placed in normal growth medium. At DIV17, transfected neurons were fixed with warm 4% PFA and 4% sucrose in PBS (pH 7.4) for 20 min and washed 3× in 1× PBS for 10 min at RT. Immunohistochemistry was performed with ADB-T (4% Normal Goat Serum, 0.1% BSA, 0.1% Triton X-100 in 1× PBS, pH 7.4) blocking buffer for 1 h at RT followed by overnight incubation with chicken anti-GFP (ab13970, Abcam) primary antibody at 4°C. The next day, three washes in 1× PBS were performed and followed by incubation with Alexa-488 conjugated goat anti-chicken secondary antibody (ab150169, Abcam) diluted in blocking buffer for 1 h at RT. Subsequently, two washes in 1× PBS were performed followed by 4’, 6-diamidino-2-phenylindole (DAPI) staining for 15 min at RT. Finally, coverslips were washed in 1× PBS and dipped in water before mounting with FluorSave™ reagent (Merck). Confocal laser microscopy (LSM880, Zeiss) was used to obtain high-resolution images with an oil-immersion 63× objective. Six to seven z-stack planes of single neurons were acquired by focusing on the closest to the soma apical dendrites. ImageJ software (version 2.1.0) cell counter plugin was used to perform dendritic spine analysis, as previously described (Vangoor et al., 2019). In summary, different spine types were categorized into five morphological classes (branched, mushroom, stubby, thin, and filopodia) and counted across an approximated dendritic length of 100 μm (Vangoor et al., 2019). Spine density was calculated by dividing the number of spines counted by dendritic length.

### RNA Isolation

RNA was collected in QIAzol lysis reagent (Qiagen) and extracted using the miRNeasy Mini kit (Qiagen) according to the manufacturer’s protocol with adjustments to each experiment.

For human hippocampal tissue, 30 μm thick cryosections were acquired until approximately 80–100 mg tissue was collected. For mouse hippocampal tissue (ICK model), the entire hippocampus was used for RNA isolation. In both cases, tissue was disrupted using a tissue homogenizer for 30 s. Before miRNeasy Mini kit steps, cells were lysed using a 1 ml 25G 5/8 precision syringe with Luer centric connection (303175, BD Plastik). For expected low-yield RNA samples (<100 ng/μl) (primary neuronal samples) isopropanol instead of EtOH was added to Buffer RWT. All RNA concentrations were determined using NanoDrop or Varioskan™ (Thermo Fisher Scientific) and stored at −80°C until use. In case of RNA sequencing (RNA-seq) samples, RNA quality was assessed using RNA 6000 Nano chip in the 2100 Bioanalyzer (Agilent).

### Library Preparation, RNA-Seq and CircRNA Analysis

CircRNA analysis was performed on RNA derived from cortical and hippocampal fractions of mTLE patients and healthy postmortem controls obtained in a previous study (Vangoor et al., 2021).

Library preparation and circRNA analysis using RNA-seq were performed as described previously (Gomes-Duarte et al., 2021), with minor modifications (human circRNA detection). Purified RNA (2 μg input) was depleted of ribosomal RNA (rRNA) using the Ribo-Zero Magnetic Kit (human/mouse/rat; Illumina). Sequencing libraries were generated using the ScriptSeq v2 kit, sequenced as paired end 100 bp reads on an Illumina HiSeq 4000 sequencer and quality checked using 2100 Bioanalyzer (Agilent). Sequencing data were preprocessed by removing adapter sequences and trimming away low quality bases (Phred score 20) with Trim Galore which uses the Cutadapt algorithm (Martin, 2011). CircRNA detection was performed by mapping filtered reads to the human genome (hg19) with Bowtie (Langmead et al., 2009), using find_circ (Memczak et al., 2013) to detect back-splice junction spanning reads from the reads that do not map linearly to the human genome. Only circRNAs with two or more supporting back-splice junction reads within single samples were kept. A second circRNA detection algorithm, CIRCexplorer (Zhang et al., 2014), was used to verify the detected circRNAs. CIRCexplorer was guided by Ensembl Release 71 gene annotations on human genome (hg19). A circRNA was defined as exonic if overlapping with one or more RefSeq exons. Correlation of circular and linear splicing was also investigated to understand whether circRNA expression changes between epileptic and control hippocampus were correlated with the linear transcript expression changes for each individual circRNA. A plot showing the log_2_ linear fold change on the x-axis and log_2_ circRNA fold change on the y-axis was made in R using ggplot with ± 1 log_2_ fold change. Differential expression analysis was done using DESeq2 (Love et al., 2014) in R. Raw counts of back-splice junction read numbers detected by find_circ were used as input for DESeq2. Statistical metrics produced by DESeq2 including probability value (*p*-value, *p*) and Benjamini-Hochberg adjusted *p*-values are reported with the normalized back-splice junction expression values generated by DESeq2. The complete list of circRNAs detected after RNA-seq and DESeq2 analysis for all samples can be found in Supplementary Table 1.

### RNase R Treatment, cDNA Synthesis and PCR

RNase R treatment, cDNA synthesis and PCR were performed as reported previously with minor modifications (Gomes-Duarte et al., 2021). RNase R treatment was performed as follows: 5 μg of total RNA was diluted in 20 μl of water with 4U RNase R/μg (Epicenter), 2 μl of enzyme buffer and then incubated for 20 min at 37°C. Reverse transcription of RNA was performed with SuperScript IV First-strand Synthesis System (Thermo Fisher Scientific): up to 5 μg of RNA were retrotranscribed in a 20 μl reaction mix according to the manufacturer’s protocol and incubated 10 min at 23°C, 10 min at 53°C and 10 min at 80°C. First, semi-quantitative PCR was used for primer validation and was performed using 500 ng cDNA with 0.3 μl of TAQ™ DNA Polymerase (Qiagen), 2 μl of 10× Reaction Buffer, 4 μl of Buffer Q and 1.6 μl of 10 μM primers in a final volume of 20 μl reaction. Reactions were carried out according to the following program: 95°C for 2 min; 34 cycles at 95°C for 30 s (specific annealing temperature) °C for 30 s, 72°C for 1 min and a final extension of 72°C for 10 min. 12 μl of each PCR reaction were run in a 1 or 2% agarose gel (m/v). Then, RT-qPCR was performed on untreated and RNAse R-treated samples according to the methodology described in the following subsection (“Reference gene selection and RT-qPCR”). In order to assess circularity, Ct differences between untreated and RNAse R-treated samples were calculated for each circular and linear RNA target. Then, the relative of expression of RNAse R-treated/untreated sample was calculated according to the 2^–ΔΔCt^ method. Proof of circularity was defined as: the fraction of the RNA recovered upon RNase R treatment regarding a certain circRNA was above the corresponding mRNA amount obtained when the same treatment was performed.

To check the homology between the human and mouse circ_Satb1 isoforms, 500 ng of cDNA derived from mouse hippocampus was used as a template in a 20 μl reaction with 2 μl of 10× Pfu DNA polymerase reaction buffer, 0.4 μl of 10 mM dNTPs, 2 μl of 10 μM primer mix (convergent primers) and 0.3 μl of Pfu DNA polymerase (Promega). PCR reaction was performed according to a touchdown method as follows: 95°C for 2 min; 34 cycles at 95°C for 1 min (62, 60, 58, and 56°C) gradient for 30 s, 72°C for 4 min and a final extension of 72°C for 5 min. 20 μl of touchdown PCR reaction was run in a 2% agarose gel (m/v), purified with PureLink Quick Gel Extraction Kit (Thermo Fisher Scientific) and analyzed using custom DNA sequencing (Standard-Seq, Macrogen).

### Reference Gene Selection and RT-qPCR

The subsequent RT-qPCR assays for mRNA and circRNA amplification were performed in a 96- or 384-well format plate in which each reaction was as follows: 5 ng of cDNA was added to a mix composed of 5 μl of 2× SYBR Mastermix (Qiagen) and 1 μl of 10 μM primer mix to a final volume of 10 μl. cDNA amplification was performed for 40 cycles at 95°C for 15 s, 60°C for 1 min and 95°C for 15 s followed by melting curve analysis. Quantitative PCR reactions were run in QuantStudio 6 flex Real-Time PCR apparatus (Applied Biosystem). All Ct values ≥35 were discarded as these were considered amplification artifacts and not reliable measurements. The 2^–ΔΔCt^ method was used in the analysis. Primers were designed using Primer 3 tool or obtained from PrimerBank^1^ and are listed in Supplementary Table 2. NormFinder (*.xla, MS Excel 2003 v0.953) was used to determine the most stable reference genes to be used considering the experimental groups analyzed (Andersen et al., 2004). NormFinder analysis focused on the intra- and inter-individual expression variation of the transcripts (Supplementary Table 3).

### Molecular Cloning

#### CircRNA Knockdown

CasRx-based technology was used to induce circRNA knockdown. Guide RNA design fulfilled the following criteria as much as possible: (a) 30 nt length, (b) maximum of four sequential nucleotide repetitions, (c) approximately 50% G/C content, and (d) as symmetric as possible across the back-splice junction. Three different guide RNAs targeting the circ_Satb1 back-splice junction were designed. 2 μg of forward and reverse oligonucleotides were resuspended in 1× annealing buffer (100 mM Tris pH = 7.5–8.0, 1 M NaCl, 10 mM EDTA, pH = 7.5) and water in a final volume of 50 μl. Annealing was performed in a heat block for 5 min at 95°C followed by slow cooling down to RT for approximately 1.5 h. The final vector for gRNA cloning was generated from the pr26 backbone (pr0026-u6-bbsi-lw2_crrnadr-puro, a gift from Feng Zhang), which was further modified. Briefly, pr0026-u6-bbsi-lw2_crrnadr-puro was first digested with *Nde*I and *Eco*RI. To generate the final destination vector (p-U6BbsI-CasRx), a synthetic dsDNA fragment containing homology arms and a CasRx gRNA direct repeat followed by two *Bbs*I restricting sites was assembled into pr0026-u6-bbsi-lw2_crrnadr-puro using NEBuilder HiFi DNA Assembly Master Mix (NEB). For gRNA cloning, 10 μg of p-U6BbsI-CasRx plasmid DNA was digested for 2 h at 95°C in a reaction containing 10× restriction buffer and *Bbs*I enzyme (NEB). Reactions were run in a 1.5% (m/v) agarose gel, excised and purified using Zymoclean™ Gel DNA Recovery Kit (Zymo Research), according to the manufacturer’s instructions. Ligation was performed at a 1:10 plasmid:insert molar ratio in a 10 μl reaction containing: 1 μl of digested p-U6BbsI-CasRx plasmid DNA, 1 μl of diluted (1:5) annealed oligonucleotides, 1 μl of T4 DNA ligase and 1 μl of 10× Buffer T4 DNA ligase. Reactions were incubated for 1 h at RT. Bacterial transformation was followed by Sanger sequencing confirmation using the U6 forward primer.

### Immunohistochemistry

#### mTLE Patient Characterization

Neuronal nuclear protein (NeuN) immunostainings were performed on resected human hippocampal tissue derived from control and mTLE individuals. Sections were fixed for 10 min in 4% PFA, washed in 1× PBS and blocked in 3% Normal Goat Serum, 0.2% Triton in 1× PBS, pH 7.4, for 1 h at RT. Incubation was performed with mouse anti-NeuN antibody (ab104224, Abcam) in blocking solution overnight at 4°C. After washing, sections were incubated with Alexa-488 conjugated donkey anti-mouse secondary antibody (A21202, Thermo Fisher Scientific) for 1.5 h at RT, followed by three washes in 1× PBS and DAPI staining. Mounting was performed using Vectashield (Vector Labs) and images were acquired with a brightfield microscope (Axio Scope A1, Zeiss).

#### Intracortical Kainic Acid Mouse Model

For the purpose of SATB1 detection in ICK mice hippocampal tissue, 16 μm thick cryosections were obtained for *n* = 3 animals per group at 30 days after Saline/KA injection. SATB1 immunostaining was performed using rabbit anti-SATB1 primary (ab49061, Abcam) and Alexa-488 conjugated goat anti-rabbit secondary antibodies. Sections were mounted using FluorSave™ reagent. Four pictures across different hippocampal regions (CA1, CA3, CA4, and DG) were taken of each animal using a confocal microscope (LSM880, Zeiss). Low objective tile scanning (5×) followed by imaging at pre-set (x, y, z) positions (20×) was performed in order to image consistent regions and avoid user bias across animals. SATB1 protein expression was determined by using the ImageJ command Analyze Particles. The average fluorescence intensity across cells and cell regions was considered for each animal. Details on antibody dilutions are listed in Supplementary Table 4.

### Single-Molecule RNA *in situ* Hybridization

Primary hippocampal neurons were fixed at DIV14 in 4% EM grade PFA (VWR) for 10 min prior to *in situ* hybridization (ISH) using the viewRNA miRNA ISH Cell Assay Kit (Invitrogen), following the manufacturer’s instructions with a few modifications. Briefly, PHN were cross-linked by two incubations in cross-linking buffer QM for 10 min, followed by a 45 min incubation in 0.16 M EDC hydrochloride. Cell membranes were permeabilized in detergent solution QC for 10 min prior to probe hybridization. Probes were diluted 1:100 in Probe set diluent QF and incubated for 3 h at 40°C in a HybEZ II hybridization oven (PN 321710, ACD). Coverslips were stored overnight at 4°C in storage buffer. For signal amplification, cells were incubated for 60 min at 40°C in Pre-Amplifier mix QM and, after washing in wash buffer, incubated in Amplifier mix QM for 1 h at 40°C. Working Label Probe mix solution was prepared by diluting label probe LP1-AP 1:1500 in Label Probe diluent QF. After brief vortexing, coverslips were transferred to a drop of working solution and incubated 60 min at 40°C. Samples were then transferred to AP Enhancer solution for 5 min at RT. AP Enhancer solution was removed completely before incubation in Fast Red substrate (1 fast red tablet in 5 ml Naphthol buffer) for 45 min at 40°C. Samples were washed two times 1 min in 1× PBS and subsequently fixed in 4% PFA for 10 min at RT.

For combining of smFISH with immunostaining, coverslips were washed three times 1 min in 1× PBS before blocking in blocking solution (5% Normal Donkey Serum, 0.5% Triton-X100, 2.5% BSA in 1× PBS) for 1 h at RT. Then coverslips were incubated with mouse anti-β-Tubulin III (T8660, Sigma-Aldrich) primary antibody diluted in blocking solution overnight at 4°C in a humidified chamber. The next day, cells were washed for three times in 1× PBS for 3 min and incubated with Alexa-647 conjugated donkey anti-mouse secondary antibody (Thermo Fisher Scientific) for 45 min at RT. After two washes in 1× PBS, DAPI staining was performed for 15 min at RT and cells washed one last time in 1× PBS. Finally, coverslips were dipped into water to remove remaining PBS and mounted in FluorSave™ reagent. Cells were imaged using a brightfield microscope. Complete details on antibody dilutions can be found in Supplementary Table 4.

### SDS-PAGE and Western Blot

Approximately 30 mg of human hippocampal tissue was homogenized in 300 μl ice-cold lysis buffer (20 mM Tris-buffer, pH 8.0, 150 mM NaCl, 0.1% glycerol, 1% Triton X-100 and complete protease inhibitor [Roche]). The protein fraction (supernatant) was collected upon centrifugation at 13,200 rpm for 15 min at 4°C. Pierce™ BCA Protein Assay Kit (Thermo Fisher Scientific) was used to determine protein concentration. Before gel application, lysates were incubated with 1× NuPAGE LDS Sample Buffer (NP0007, Invitrogen), 10% β-mercaptoethanol (805740, Merck), and boiled for 10 min at 95°C. 20 μg lysate was resolved in a 10% SDS-PAGE gel (Acrylamide/Bis-acrylamide solution [1610156, BioRad], 0.75 M Tris, pH 8.8, 1% SDS, 1% APS and TEMED [110732, Merck]) at 100–150 V. Then, proteins were transferred onto a 0.2 μm nitrocellulose membrane (Invitrogen) in cold 1× Transfer Buffer (100 ml 10× SDS-PAGE buffer, 200 and 700 ml water), for 1 h at 100 V. Following transfer, membranes were blocked with blocking buffer (5% ELK in 1× Tris-buffered saline-Tween 20 [TBS-T]) for 1 h at RT. Primary antibody incubation was performed at 4°C overnight in a wet chamber using rabbit anti-SATB1 (ab49061, Abcam) or mouse anti-β-actin (A5316, Sigma-Aldrich) antibodies, both diluted in blocking buffer. The following day, membranes were washed in 1× TBS-T for three times, each 10 min. After this, secondary antibody incubation was performed in modified blocking buffer (2.5% ELK in 1× TBS-T) for 1 h on a shaker at RT using goat anti-rabbit (111-035-003, Jackson ImmunoResearch Europe Ltd.) or goat anti-mouse (115-036-020, Jackson ImmunoResearch Europe Ltd.) horseradish peroxidase (HRP)-conjugated antibodies. Membranes were finally washed in 1× TBS-T for three times, each 10 min. Developing reaction was performed using the SuperSignal West Dura Extended Duration Substrate Kit (34075, Thermo Fisher Scientific), according to manufacturer’s instructions. Images were acquired using a FluorChem scanner (ProteinSimple) at variable exposure times (30 s–10 min).

Protein levels were quantified by measuring band intensity using ImageJ software. The rabbit anti-SATB1 antibody detects two described SATB1 isoforms produced by alternative splicing (UniProtKB – Q01826). SATB1 expression levels were normalized against β-actin. For complete details on antibody dilutions see Supplementary Table 4.

### Pearson’s Correlation and Gene Ontology Analysis

Pearson’s correlation coefficient (r) was used to assess linear relations between (1) expression levels of circ_Satb1/2 and deregulated mRNA transcripts in mTLE patients, and (2) expression levels of circ_Satb1 and relevant clinical information of mTLE patients.

For (1), expression of circ_Satb1/2 as detected from the RNA-seq data (CIRCexplorer quantification) was RPM normalized, log2 transformed and correlated with the TPM normalized log2 transformed expression level of genes significantly downregulated in the nuclear fraction of hippocampal tissue. The correlation was computed in R using the cor() function and plotted using the pheatmap package. Gene ontology (GO) analysis of selected targets was performed using the Gene Ontology enRIchment anaLysis and visuaLizAtion (GOrilla) tool^2^ (Eden et al., 2009). A running mode including two unranked lists of genes (target and background lists) was used. The background list included all nuclear downregulated transcripts (genes) whereas the target list included all nuclear transcripts (genes) found to be positively co-expressed with circ_Satb1. The enrichment score associated with each GO term was calculated as the negative logarithm of the *p*-value (p) (−log_10_p). For (2), expression level of circ_Satb1/2, as evaluated by RT-qPCR for individual patients, was correlated with the following patient disease metrics: Age of onset, Age at surgery, Seizure frequency and Seizure-free period. The correlation was computed as described above and plotted using the ComplexHeatmap package.

### Statistical Analysis

Statistical analysis was performed using GraphPad Prism version 8.4.2 (GraphPad Software, San Diego, CA, United States).

Expression levels of linear and circular RNA transcripts were analyzed for control and patient samples using unpaired two-tailed Student’s *t*-test with Welch’s correction. To compare RNA expression changes across the ICK model time points, multiple unpaired *t*-tests with Šídák’s test for multiple comparisons were used; *n* = 3–5 animals per group.

Regulation of circRNA expression upon depolarization with KCl or Mg^2+^ depletion was statistically assessed using an ordinary one-way ANOVA without correction for multiple comparisons (Fisher’s LSD test) or multiple unpaired *t*-tests, respectively; *n* = 3–5 independent experiments. Efficiency of circRNA knockdown constructs was analyzed statistically using an unpaired two-tailed Student’s *t*-test; *n* = 2–3 independent transfections.

Protein expression in human samples and cell lysates were assessed by measuring the density of bands detected by Western Blot. Protein expression was determined by normalizing the density of the bands of the protein of interest to the ones of a standard protein. *N* = 6 individuals per group were used in both control and patient samples. To evaluate SATB1 protein expression in the ICK model, SATB1 expression was measured using particle analysis (threshold method and size [μm^2^] = 20-Infinity) in ImageJ. Nuclear expression was defined as the average fluorescence intensity for each animal, across different hippocampal regions. An average of 43 cells was measured for each animal (*N* = 3 per group). Statistical analysis of protein changes was performed using an unpaired two-tailed Mann-Whitney test.

For functional assays, PHN were used and three independent transfections performed. Spine density was quantified between groups using unpaired Student’s *t*-test. Statistical analysis of spine morphology was performed using two-way ANOVA for each comparison and *post hoc* Fisher’s LSD test. A total of 36 neurons was analyzed across conditions and experiments.

For *in vivo* animal experiments (ICK mouse model), sample size was estimated using an online statistical tool^3^, with Power = 0.8 and Alpha = 0.05, based on the effect size observed for ncRNAs in animal models of TLE in published literature (Jimenez-Mateos et al., 2012). In the remaining experiments, no statistical methods were applied to predetermine the n to be used but sample size resembled that generally used in the field. All values are expressed as mean ± the standard error of the mean (SEM) or standard deviation (SD). Significance was considered for *p* < 0.05 (*p* < 0.0001: ^****^; *p* < 0.001: ^***^; *p* < 0.01: ^**^; *p* < 0.05: *; *p* ≥ 0.05: ns: not significant) in all statistical tests with the exception of GO analysis with GOrilla, where *p* < 0.01 was set as the threshold.

## Results

### The Human Hippocampus Expresses Thousands of Exonic CircRNAs

**FIGURE 1:**
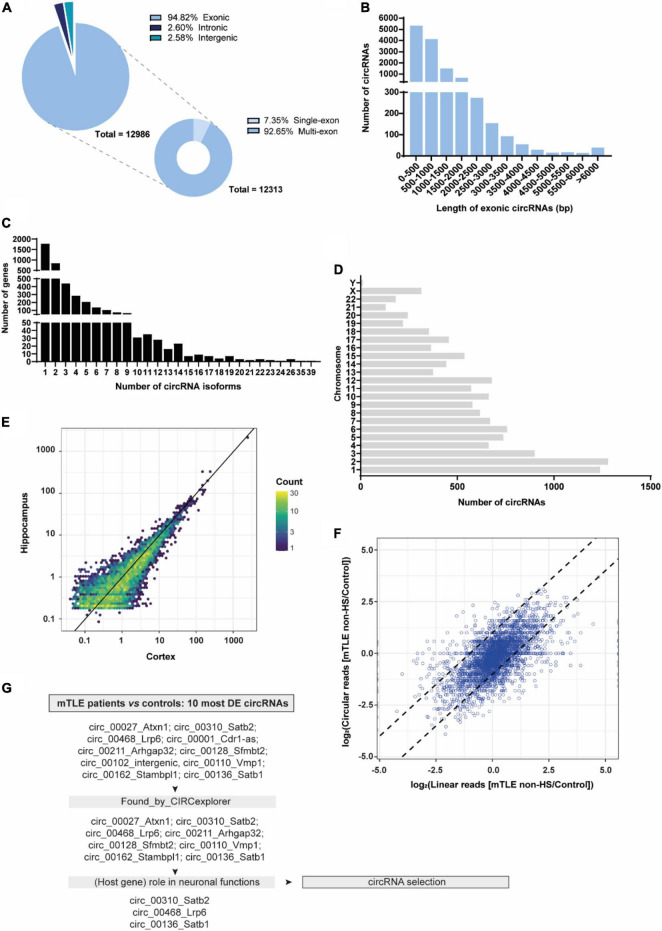
CircRNA profiling of autopsy controls and mTLE patients. **(A)** RNA composition of all detected circRNAs (exonic, intronic, and intergenic). **(B)** Exonic length distribution of detected circRNAs. *N* = 12313 (bp, base pairs). **(C)** Distribution of circRNA isoforms across detected circRNAs from both cortex and hippocampus. *N* = 12986, of which *n* = 11914 were detected in the cortex and *n* = 6947 in the hippocampus. Number of circRNA isoforms were calculated per host gene. **(D)** Distribution of detected circRNAs across human chromosomes. **(E)** Scatter plot representing the mean normalized expression (nucleus and cytoplasm) of circRNAs in the cortex (*x*-axis) and hippocampus (*y*-axis). Color scale indicates the number of overlapping circRNAs. Expression values were log_10_ transformed to allow visualization of all circRNAs. **(F)** Expression correlation of circular and linear RNA splicing in hippocampal samples. The plot shows the log2 linear fold change on the *x*-axis and log2 circRNA fold change on the *y*-axis between mTLE non-HS and control samples, ±1 log2 fold change is indicated by the dashed lines. CircRNAs with expression correlated to the host gene linear transcript are in the central diagonal and non-correlated circRNAs are depicted outside of the diagonal. **(G)** Schematic workflow representation of the pipeline used to select circRNAs of interest from the 10 most DE circRNAs found after DESeq2 analysis and Benjamini-Hochberg testing correction.

To determine the circRNA transcriptome of mTLE patients, RNA was collected from the cortex and hippocampus of mTLE patients (with or without hippocampal sclerosis [HS]) and healthy postmortem controls and subjected to RNA-seq (Vangoor et al., 2021). High-throughput sequencing identified a total of 12986 circRNAs (≥two or more back-splice junction reads detected) across control and mTLE groups of both cortex and hippocampal regions and cellular compartments. Among the two regions, 11914 circRNAs were detected in the cortex and 6947 circRNAs in the hippocampus. The majority of these circRNAs was exonic (94.82%), while others were classified as intronic (2.60%) or intergenic (2.58%). When considering exonic circRNAs (*n* = 12313), a strong enrichment for multi-exon circRNAs was observed (92.65%), whereas only few circRNAs (7.35%) were composed by a single exon (Figure 1A). CircRNAs are commonly 100–400 bp long (Lasda and Parker, 2014). In line with this, the predicted length of the vast majority of exonic circRNAs detected (76.71%) was below 1000 bp (Figure 1B). Furthermore, the number of circRNA isoforms showed a skewed distribution, with most genes generating one or two circRNA isoforms (63.55%) (Figure 1C). Detected circRNAs originated from genes distributed across most human chromosomes with the exception of the Y chromosome, with only 11 circRNAs produced. This observation is in line with previous profiling studies using epileptic human tissue and animal models of epilepsy (Li et al., 2018; Gomes-Duarte et al., 2021) and may derive from the low gene density that characterizes the Y chromosome (Lluís and Fellous, 2001; Ovcharenko et al., 2005; Figure 1D). Regarding abundance, circRNA expression was heterogeneous and, to some extent, tissue-specific, with some circRNAs being expressed almost exclusively in either the cortex or hippocampus (Figure 1E). Furthermore, our analysis revealed that, for the majority of genes, expression of circRNAs correlated with the expression of their host mRNA in mTLE non-HS. However, for some circRNAs, expression changes appeared to be independent of their linear counterparts indicating deregulation of circRNA expression is not always coupled with host gene expression (Figure 1F).

Next, a few candidates were selected from the RNA-seq dataset to further investigate circRNA expression and function in relation to mTLE. DESeq2 analysis was performed on both cortex and hippocampus tissue from mTLE patients and controls and yielded 333 differentially expressed (DE) circRNAs between healthy individual and mTLE patient samples. Of these, 23 circRNAs had significant adjusted *p*-values after application of the Benjamini-Hochberg procedure for multiple testing correction. In order to select circRNAs for subsequent studies, we applied a three-step pipeline. First, we selected the 10 most DE circRNAs based on adjusted *p*-values (Table 2). Second, we excluded circRNAs in which back-splice sites identified by find_circ (Memczak et al., 2013) were not confirmed by CIRCexplorer (Zhang et al., 2014). After this selection, most of the selected circRNAs shared multiple exons with their host genes (Supplementary Table 1). Based on this sequence similarity we predicted that circRNAs may share some of the functions of their host genes and we used the reported neuronal roles of host genes as a third criterion. For example, special AT-rich sequence-binding protein 2 (Satb2) is required for the development of cortical connections (Alcamo et al., 2008) and loss of Satb2 leads to memory dysfunction and hyperactivity (Zhang et al., 2019). Similar defects in synaptic function and memory have been reported as a consequence of LDL Receptor Related Protein 6 (LRP6) dysfunction (Liu et al., 2014). Satb1 loss in mice leads to an impaired excitation/inhibition balance in the cortex and dendritic spine changes (Balamotis et al., 2012; Close et al., 2012). Also, Satb1 plays a role in promoting differentiation, connectivity and survival of cortical parvalbumin and somatostatin interneurons (Close et al., 2012). Comparison of these results with pathogenic hallmarks of epilepsy reveals an interesting overlap and highlights circ_Satb2, circ_Lrp6 and circ_Satb1 as interesting candidates in relation to mTLE (Jarero-Basulto et al., 2018).

**TABLE 2:** List of the 10 most DE circRNAs in the hippocampus of mTLE non-HS patients in comparison to healthy postmortem controls.

CircRNA_name	Genomic coordinates (hg19)	circBase_ID	Adjusted *p*-value	log2FC nuc	log2FC cyt	Host gene description
circ_00027_ATXN1	chr6:16326624-16328701	hsa_circ_0007132	1,22E-04	5, 48	−0, 21	*Ataxin 1*
circ_00310_SATB2	chr2:200173482-200246543	hsa_circ_0006011	9,32E-03	−0, 49	−5, 63	*Special AT-rich sequence-binding protein 2*
circ_00468_LRP6	chr12:12397195-12397589	hsa_circ_0000378	9,32E-03	1, 65	−6, 73	*Low-density lipoprotein receptor-related protein 6*
circ_00001_CDR1-AS	chrX:139865339-139866824	hsa_circ_0001946	1,35E-02	−0, 37	−0, 71	*Cerebellum degeneration-related antigen 1 (antisense)*
circ_00211_ARHGAP32	chr11:128993340-129034322	hsa_circ_0007843	1,91E-02	1, 68	−0, 30	*Rho GTPase Activating Protein 32*
circ_00128_SFMBT2	chr10:7318853-7327916	hsa_circ_0000211	1,21E-02	4, 96	0, 75	*Scm Like With Four Mbt Domains 2*
circ_00102_intergenic	chr18:44526019-44526886	hsa_circ_0108513	1,35E-02	2, 64	0, 05	-
circ_00110_VMP1	chr17:57808781-57851246	hsa_circ_0005077	3,86E-02	1, 52	−0, 40	*Vacuole Membrane Protein 1*
circ_00162_STAMBPL1	chr10:90661412-90682193	hsa_circ_0019061	1,60E-02	1, 61	−0, 51	*STAM Binding Protein Like 1*
circ_00136_SATB1	chr3:18419661-18462483	hsa_circ_0064555	1,79E-02	−4, 36	−0, 35	*Special AT-rich sequence-binding protein 1*

*hg19, Homo sapiens (human) genome assembly GRCh37; FC, fold-change; nuc, nucleus; cyt, cytoplasm.*

In summary, we applied the following circRNA selection criteria: (1) 10 most DE circRNAs between mTLE patients and control samples, (2) detection by CIRCexplorer, (3) role (of host gene) in neuronal functions (Figure 1G). This selection yielded three circRNAs for further study: circ_Satb1, circ_Satb2 and circ_Lrp6.

### Downregulation of CircRNAs in the Mesial Temporal Lobe Epilepsy Hippocampus

**FIGURE 2:**
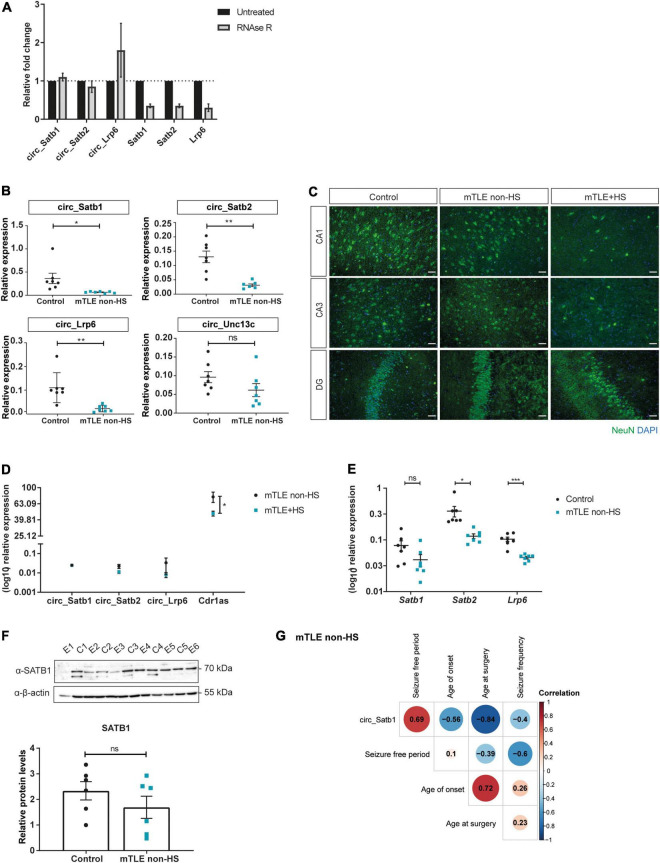
CircRNA deregulation in mTLE patients. **(A)** Quantification of circular and linear RNA products amplified by RT-qPCR following treatment with RNase R. Values are expressed as fold change means ± SD and are normalized to the untreated condition. *N* = 2 independent experiments. **(B)** RT-qPCR analysis of the expression pattern of selected circRNAs in autopsy controls and mTLE non-HS patients (circ_Satb1, *p = 0.0364; circ_Satb2, **p = 0.0036; circ_Lrp6, **p = 0.0096; circ_Unc13c, ns). Each dot represents one individual and shows circRNA expression relative to RNA-polymerase II (*RPII)* and TATA-Box Binding Protein (*TBP*) transcripts. Values are expressed as means ± SEM. Unpaired two-tailed *t*-test with Welch correction; *n* = 7 individuals per group. **(C)** Characterization of mTLE (non-HS and +HS) and control tissue. Neuronal nuclei (NeuN) staining was performed to characterize distinct mTLE subtypes, with (+HS) or without (non-HS) hippocampal sclerosis, in comparison to autopsy control. NeuN staining is represented in the green channel (488) and 4,6-diamidino-2-phenylindole (DAPI) in the blue channel (405). Scale bar: 50 μm (mTLE, Mesial Temporal lobe epilepsy; HS, hippocampal sclerosis; CA1/3, Cornu Ammonis area 1/3; DG, dentate gyrus). **(D)** RT-qPCR analysis of the expression pattern of selected circRNAs in mTLE patients with (+HS) and without (non-HS) HS (circ_Satb1, circ_Satb2, and circ_Lrp6, ns; Cdr1as, *p = 0.0387). Dot plots represent circRNA expression relative to *RPII* and *TBP.* Values are expressed as means ± SEM and were log_10_ transformed to allow for proper visualization and further statistical analysis, considering the spread of the data. Multiple unpaired *t*-tests; *n* = 4–5 individuals per group. **(E)** RT-qPCR analysis of the expression pattern of circRNA linear counterparts in autopsy controls and mTLE non-HS patients (*Satb1*, ns; *Satb2*, *p = 0.0290; *Lrp6*, ***p = 0.0003). Each dot represents one individual and shows mRNA expression relative to *RPII* and *TBP.* Values are expressed as means ± SEM and were log_10_ transformed to allow for proper visualization and further statistical analysis, considering the spread of the data. Unpaired two-tailed *t*-test with Welch correction; *n* = 7 individuals per group. **(F)** SATB1 protein levels in autopsy controls and mTLE non-HS patients, normalized to β-actin. The upper panel shows SATB1/β-actin detection following SDS-PAGE and Western blot. In the lower panel, bar plots represent mean ± SEM and each dot a specific individual, *p* ≥ 0.05: ns. Unpaired two-tailed Mann-Whitney test; *n* = 6 individuals per group (E, epilepsy; C, control). **(G)** Correlation matrix heatmap of circ_Satb1 expression and clinical information of mTLE non-HS patients. Pearson correlation values are represented in each cell. *N* = 7 individuals per group.

CircRNAs are usually more resistant to exonuclease degradation as compared to their linear counterparts (Suzuki et al., 2006). To assess circularity of the selected circRNAs, RNase R treatment was performed on human hippocampal control tissue. In all cases, the fraction of RNA recovered upon RNase R treatment was higher for circular as compared to linear transcripts, confirming the circular nature of the selected candidates (Figure 2A).

Our study determined the circRNA transcriptome of hippocampal and cortical tissue. Deregulation of all three selected candidates was similar in both tissue types. Since the origin of seizure onset in mTLE is often hippocampal (Tatum, 2012), we focused on circRNA changes in hippocampal tissue. We validated the observed RNA-seq changes in an independent set of samples from mTLE patients (non-HS) and postmortem controls (*n* = 7). In line with the RNA-seq data, downregulation of circ_Satb1, circ_Satb2 and circ_Lrp6 was observed in the hippocampus of mTLE patients in comparison to controls (Figure 2B). Levels of circ_Unc13c (hsa_circ_0103896), selected as a non-DE circRNA from the RNA-seq dataset, were similar in controls and mTLE patients. CircRNA accumulation is a characteristic of the aging brain (Gruner et al., 2016). Therefore, the observed downregulation could result from the older age of the postmortem tissue (and thus more accumulation). However, no clear association was found between age and expression of selected (and additional) circRNAs (Supplementary Figure 1). Since hippocampal sclerosis (HS), characterized by severe neuron loss and gliosis, is a hallmark of a subset of mTLE patients (Blümcke et al., 2013), we also investigated whether circRNA expression was altered in the presence of HS (Figure 2C). However, no differences in the expression of circ_Satb1, circ_Satb2 and circ_Lrp6 were found between patients with or without HS (Figure 2D). Interestingly, expression of Cdr1as, which was included as a positive control, was more robustly decreased in mTLE patients with HS. Next, we assessed whether the linear counterparts of the selected circRNAs were also downregulated and found *Satb2* and *Lrp6* expression to be decreased in mTLE non-HS patients in comparison to controls (Figure 2E). *Satb1* expression was not significantly changed, suggesting that *Satb1* changes are specific to the circular transcript. To confirm this, SATB1 protein levels were assessed in controls and mTLE non-HS patients. No significant change in SATB1 protein was observed (Figure 2F). Of note, the presence of a specific SATB1 isoform was observed almost exclusively in control samples (Figure 2F). However, given the postmortem identity of the control group and how alternative splicing events can be altered in the postmortem human brain (Labadorf and Myers, 2015), we decided to focus on the primary SATB1 (70KDa) band only. Together, these results revealed circRNA-specific deregulation in the *Satb1* locus.

To begin to assess the potential effects of circ_Satb1 deregulation in mTLE, circ_Satb1 expression was correlated with mTLE clinical features (Figure 2G). Circ_Satb1 expression was found to be positively correlated with the seizure-free postoperative period in mTLE non-HS (*r* = +0.7). As circ_Satb1 expression is markedly decreased in mTLE patients, these results suggest that high circ_Satb1 expression may be a predictor of a positive postoperative outcome. Circ_Satb1 expression was negatively correlated with age at surgery (*r* = −0.8) and age of onset (*r* = −0.6). No significant associations were found between circ_Satb1 expression and seizure frequency and duration in mTLE non-HS, or with overall clinical features in mTLE + HS (Supplementary Table 5). Also, no correlation was observed between circ_Satb2 expression and clinical aspects of mTLE (Supplementary Table 5). Together, these results show mTLE-associated downregulation of specific circRNAs and correlation of some of these changes to mTLE clinical features.

### Decreased Circ_Satb1 at the Chronic Stage of Experimental Epilepsy

**FIGURE 3:**
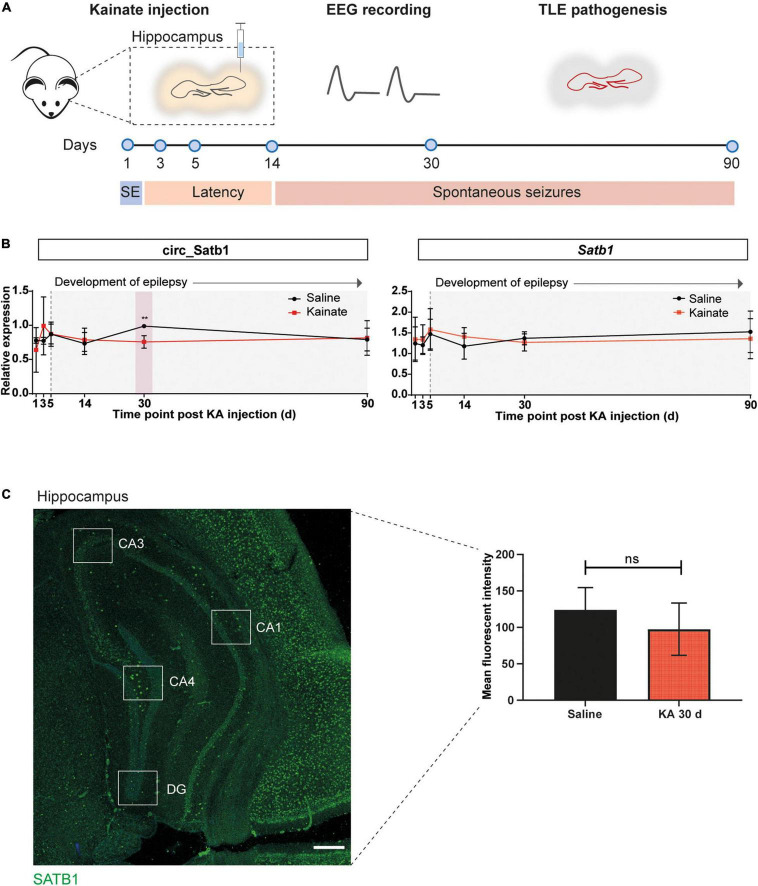
Deregulation of circ_Satb1 in a mouse model of TLE. **(A)** Schematic representation of the intracortical kainate (ICK) model of TLE used in the present study. Kainate or saline were injected unilaterally into the neocortex above right dorsal hippocampus, and the animals were followed over a period of 90 days (d) with electroencephalogram (EEG) recordings. Hippocampal samples were collected at six different time points post-injection: 1 day (*status epilepticus*, SE), 3 days after SE; 5 and 14 days (latent period); 30 and 90 days (chronic spontaneous seizures). **(B)** *Satb1*/circ_Satb1 expression relative to *Phosphoglycerate kinase* (*Pgk*) in the ICK model of TLE at different time points (circ_Satb1 at KA 30 days, **p = 0.0062). Dot plots represent means ± SEM. Multiple unpaired *t*-tests with Holm-Šídák method for multiple comparisons; *n* = 3–6 animals per group (KA, kainate/kainic acid; d, days). **(C)** SATB1 protein levels in the hippocampus of saline- and KA-injected (30 days) mice. Expression levels were calculated as the fluorescence intensity across five well-defined hippocampal regions for each animal (illustrative image, left panel). Bar plots express group mean ± SEM. Unpaired two-tailed Mann-Whitney test; *n* = 3 animals per group (right panel). Scale bar: 500 μm (CA1–4, Cornu Ammonis area 1–4; DG, dentate gyrus).

The specific deregulation of circ_Satb1 in human mTLE and its correlation to several mTLE clinical features, prompted us to investigate circ_Satb1 and *Satb1* expression in experimental mTLE. An ICK mouse model was used to examine expression at different time points post-KA injection (1, 3, 5, 14, 30, and 90 days). Kainate is a glutamate receptor agonist which provokes spontaneous recurrent seizures eventually leading to chronic epilepsy (Bedner et al., 2015). The ICK model replicates mTLE pathophysiology, from post-KA injection (including SE) (1–3 days), through the latent period (3–14 days), to spontaneous seizures (14–90 days). It shows the main pathological hallmarks of human mTLE, including neuronal death, gliosis, granule cell dispersion and astrocytic dysfunction (Bedner et al., 2015; Binder and Steinhäuser, 2017; Figure 3A and Supplementary Figure 2).

Circ_Satb1 is composed of eight exons and is generated from back-splicing and covalent binding between exons 9 and 2 (Supplementary Table 1). Human and mouse circ_Satb1 sequences are highly similar (93%) (Supplementary Figure 3A). As the mouse circ_Satb1 isoform was not previously retrieved by RNA-seq, we performed custom DNA sequencing of the mmu_circ_0006823 back-splice junction to confirm the existence of this circRNA. Back-splice junction sequencing of mmu_circ_0006823 in the mouse hippocampus revealed a perfect match to circBase annotations and parts of the *Satb1* transcript (*ENSMUST00000144331*) (Supplementary Figure 3B). In addition, it confirmed that mouse and human circ_Satb1 are formed by similar back-splicing events involving exons 9 and 2. Quantitative PCR on hippocampal tissue of ICK mice showed a small decrease in circ_Satb1, but not *Satb1*, expression at 30 days post-KA injection, in comparison to saline-injected mice (Figure 3B). No differences were observed at other time points. We next assessed SATB1 protein levels in the hippocampus of KA-injected mice at 30 days post-injection by immunostaining. In line with the results for mTLE non-HS patients, no difference in SATB1 expression was detected between control and experimental TLE mice (Figure 3C). Together, these studies identify decreased circ_Satb1 expression as a conserved disease mechanism at chronic stages of human and experimental TLE.

### Specific Spatiotemporal Patterns of Neural Circ_Satb1 Expression

**FIGURE 4:**
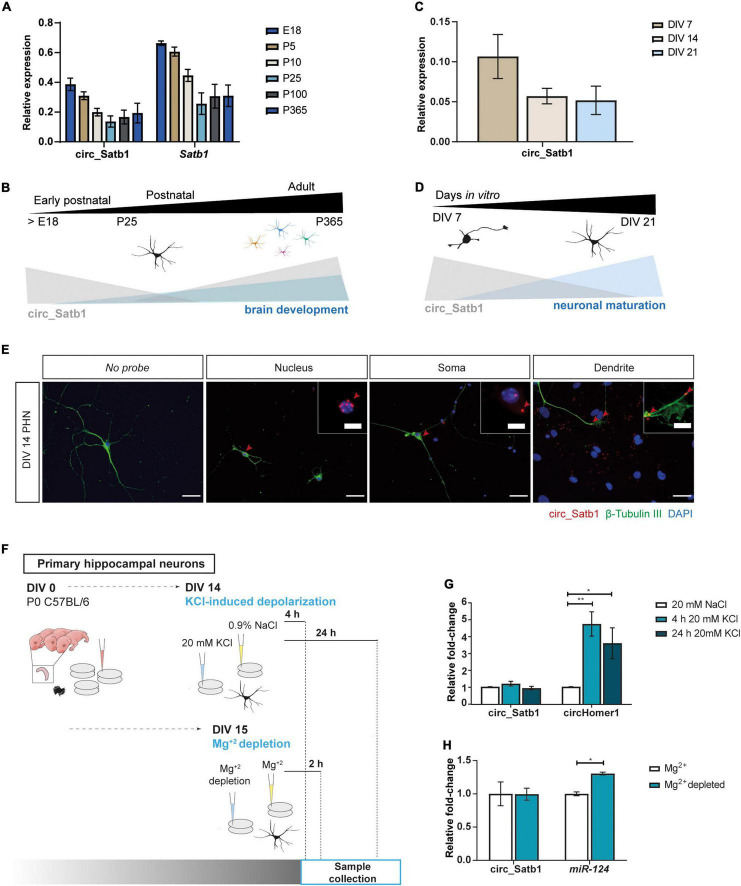
Characterization of neural circ_Satb1 expression. **(A)** Expression profile of *Satb1*/circ_Satb1 in the developing hippocampus, normalized to *Pgk.* Bar plots show group mean ± SD. *N* = 3 animals per time point (E, embryonic; P, postnatal). **(B)** Schematic representation of the circ_Satb1 profile during neuronal maturation. **(C)** Expression profile of circ_Satb1 in mouse PHN at indicated time points, normalized to *Pgk.* Bar plots express group mean ± SD. *N* = 2 independent experiments (DIV, day *in vitro*). **(D)** Schematic representation of the circ_Satb1 profile during postnatal development of the hippocampus. **(E)** Detection of circ_Satb1 with fluorescent *in situ* hybridization in PHN (red). Neurons were stained with neuron-specific class III beta-tubulin (β-Tubulin III) (green) and nuclei with 4,6-diamidino-2-phenylindole (DAPI) (blue). Scale bar: 40 μm. Red arrows indicate circ_Satb1 relevant subcellular localization signals (nucleus, soma, and dendrites), which are shown at higher magnification in upper right panel images. Scale bar: 10 μm. **(F)** Schematic overview of the functional assays performed in PHN. **(G)** Circ_Satb1 fold-change upon KCl treatment of PHN, normalized to *Gapdh*. Bar plots represent fold-changes relative to control condition (NaCl) ± SEM. CircHomer1 was used as a positive control (You et al., 2015) (**p = 0.0078, *p = 0.0348). Ordinary one-way ANOVA; *n* = 3 independent experiments (NaCl, saline; KCl, potassium chloride; *Gapdh*, *Glyceraldehyde 3-phosphate dehydrogenase*). **(H)** Circ_Satb1 and *miR-124* fold-change upon extracellular Mg^+2^ depletion of PHN neurons, normalized to *Gapdh* or *5s* rRNA. Bar plots represent fold-changes relative to control condition (Mg^+2^) ± SEM. *MiR-124* was used as a positive control (*p = 0.0020), as a highly enriched miRNA in neuron-derived exosomes (Jiang et al., 2020). Multiple unpaired *t*-tests with Holm-Šídák method for multiple comparisons; *n* = 3 independent experiments (Mg^+2^, magnesium).

The reduced expression of circ_Satb1 in mTLE patients and experimental TLE hints at a role for this circRNA in the pathogenic process underlying this disease. As the functional role of circ_Satb1 is unknown, we first studied *Satb1* and circ_Satb1 expression during mouse hippocampus development, from late embryonic (E18) to adult stages (P365) (Figure 4A). Circ_Satb1 and *Satb1* displayed a similar pattern of temporal expression, although circ_Satb1 levels were lower as compared to *Satb1*. The pattern of circ_Satb1 expression was inversely correlated with hippocampal development at early postnatal stages, and hinted at enrichment at adult stages (Figure 4B). Expression of circ_Satb1 was also assessed in primary hippocampal neurons (PHN) and shown to decrease as cultures matured (Figures 4C,D). Based on this analysis we employed PHN to study circ_Satb1 in more detail. Single-molecule fluorescent *in situ* hybridization (smFISH) experiments confirmed the expression of circ_Satb1 in PHN, as indicated by the co-localization of circ_Satb1 and β-Tubulin III (Figure 4E and Supplementary Figure 3C). Further, smFISH localized circ_Satb1 to perinuclear regions and soma compartments and, to a lesser extent, dendrites at DIV14 (Figure 4E).

Previous studies have reported circRNA regulation in response to neuronal activity (Rybak-Wolf et al., 2014; You et al., 2015; Xu et al., 2018). Interestingly, synchronized neuronal activity and altered neurotransmission are seizure-causing triggers (Dalby and Mody, 2001). Therefore, two different assays were performed to assess the effect of enhanced neuronal activity in PHN on circ_Satb1 expression (Figure 4F). Synaptic activity, neurotransmission and excitotoxicity are regulated through increased intracellular Ca^2+^ via L-type Ca^2+^ channels, which can be activated with KCl (Bading et al., 1993). KCl treatment of PHN did not influence circ_Satb1 expression at 4 or 24 h post-stimulation, in contrast to strong regulation of circHomer1 (positive control; You et al., 2015; Figure 4G). Mg^+2^ is a potent modulator of epileptic activity via its ability to regulate N-methyl-d-aspartate (NMDA) receptors (Chahal et al., 1998). Low extracellular Mg^+2^ has been linked to epileptiform activity in hippocampal neurons and slices (Walther et al., 1986; Mody et al., 1987; Sombati and DeLorenzo, 1995). Similar to the observations from the KCl assay, no changes in circ_Satb1 expression were detected following 2 h exposure to Mg^+2^-depleted culture medium, while a mild increase in *miR-124* expression was observed (Figure 4H). In summary, these results show that *in vitro* induction of neuronal activity does not alter the expression of circ_Satb1.

### Downregulation of Circ_Satb1 Causes Dendritic Spine Defects

**FIGURE 5:**
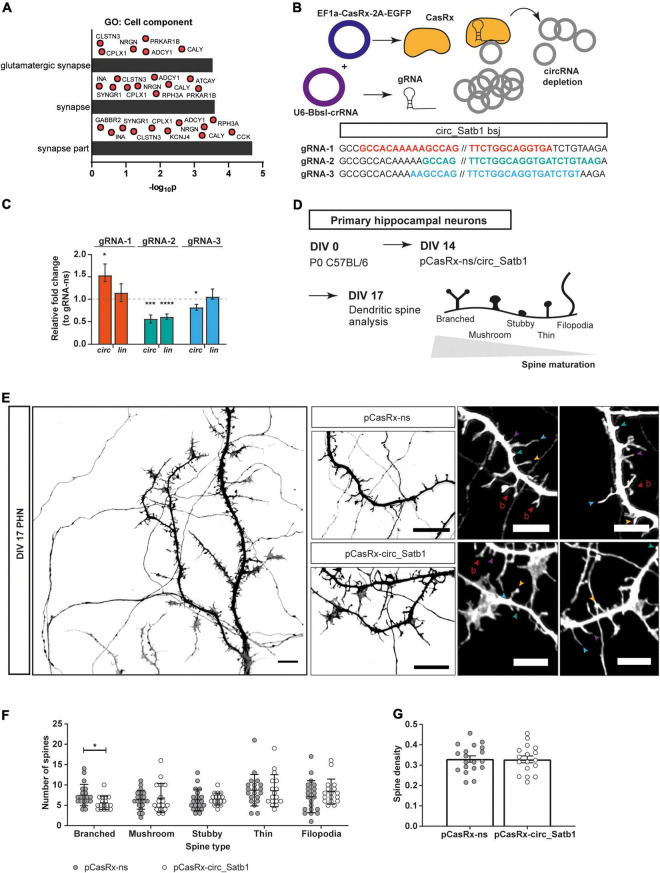
Circ_Satb1 downregulation affects spine morphology. **(A)** Gene ontology (GO) analysis of circ_Satb1 co-expressed transcripts in mTLE hippocampal samples. GO category “Cell component” is shown (*p* < 0.01) (*p*, *p*-value). **(B)** CasRx-based knockdown system and design. Three guide RNAs (gRNA) targeting different sites across the back-splice junction were designed and cloned into the U6-*Bbs*I-crRNA vector. **(C)** Validation of circ_Satb1 knockdown constructs (gRNA-1–3), 48 h post-transfection in Neuro-2a cells. Bar plots show transcript expression normalized to *Hprt1* and relative to control condition (gRNA-ns, dotted line) ± SEM; *p = 0.0411, ***p = 0.0009, ****p < 0.0001, *p = 0.0346. Unpaired two-tailed *t*-test; *n* = 4–6, 2–3 independent transfections (ns, non-specific; *circ*, circular; *lin*, linear; *Hprt1, Hypoxanthine Phosphoribosyltransferase 1*). **(D)** Workflow schematics of dendritic spine analysis experiments. PHN were cultured and transfected with Cas-Rx knockdown constructs or control vectors at days *in vitro* (DIV) 14. Neurons were fixed at DIV17 and the dendritic spines were analyzed after image acquisition using confocal microscopy. **(E)** Representative images of dendritic spines present in pCasRx-ns and pCasRx-circ_Satb1 conditions. Signal shows GFP detection (pCasRx_EGFP) in a single DIV17 neuron at 63× magnification and selected dendritic regions transfected with different CasRx-constructs. Images were converted to gray scale for improved visualization. Scale bar: 10 μm. Right side panel shows differences in spine morphology between pCasRx-ns and pCasRx-circ_Satb1 transfected neurons. Arrows indicate different spine morphology types, classified as reported previously (Vangoor et al., 2019): filopodia (blue); thin (purple); stubby (green); mushroom (yellow); and branched (red). Scale bar: 5 μm. **(F)** Spine morphology distribution upon circ_Satb1 knockdown in neuronal cultures. Bars represent mean ± SD, *p = 0.0112. Each dot represents a single analyzed neuron. Two-way ANOVA (*F* statistic = 4.548; *p* = 0.0049) with Fisher’s LSD *post hoc* test (*p* < 0.05); *n* = 3 independent cultures. **(G)** Quantification of spine density upon circ_Satb1 knockdown in neuronal cultures. Bar plots represent mean ± SEM. Each dot represents a single analyzed neuron. *N* = 3 independent cultures.

One of the reported mechanisms-of-action of circRNAs is regulation of gene expression, e.g., via miRNA or RBP binding, or competition with canonical splicing (Kristensen et al., 2019). Therefore, transcripts co-expressed with circ_Satb1 are interesting when trying to dissect the role of this RNA in mTLE pathogenesis. Pearson correlation analysis was performed between the expression of circ_Satb1 and mRNA transcripts in the hippocampus of mTLE patients. Five different datasets were considered: up- and downregulated genes detected by RNA-seq in nuclear or cytoplasmic fractions (Vangoor et al., 2021), and mRNAs from 84 core-epilepsy genes (Wang et al., 2017). The strongest correlation clustering in mTLE hippocampal samples was observed between circ_Satb1 expression and transcripts downregulated in the nuclear compartment and subsequent analysis therefore focused on associations between circ_Satb1 expression and downregulated transcripts in the nucleus (Supplementary Figure 4). Of these, 38 transcripts were positively co-expressed and 52 negatively co-expressed with circ_Satb1 in the mTLE hippocampus (Supplementary Table 6). Also, several of these transcripts were co-expressed with each other. This may indicate the presence of transcript association networks that participate in the same biological processes. Therefore, GO analysis was performed on circ_Satb1 co-expressed transcripts (Supplementary Table 7). The significant GO terms retrieved for positively co-expressed circ_Satb1 targets related to the synapse (“glutamatergic synapse,” “synapse,” and “synapse part”) (Figure 5A). No enrichment was found for negatively correlated transcripts.

A plethora of studies using mTLE tissue and experimental epilepsy models have consistently reported synaptic defects in hippocampal and neocortical pyramidal neurons, including dendritic spine loss and altered dendritic morphology (Swann et al., 2000; Wong, 2005; Wong and Guo, 2013). To investigate the effect of reduced circ_Satb1 expression, as observed in mTLE, on dendritic morphology, we used the CRISPR-CasRx system to induce knockdown of circ_Satb1 *in vitro* (Konermann et al., 2018). First, we designed three 25–28 nt guide RNAs (gRNAs) targeting the circ_Satb1 back-splice junction that differed in: (1) symmetry across the back-splice junction, and (2) potential binding to linear *Satb1* mRNA (Wessels et al., 2020; Supplementary Figure 5A). Next, we combined two constructs, one for the CasRx protein (EF1a-CasRx-2A-EGFP) and one for each gRNAs (p-U6BbsI-CasRx 1–3), to perform knockdown in Neuro-2a cells (Figure 5B). Using this approach, gRNA-1 induced circ_Satb1 upregulation, and gRNA-2/3 led to a decrease of circ_Satb1 expression in comparison to a non-specific (ns) gRNA construct (Figure 5C). However, gRNA-2 targeted both circ_Satb1 and *Satb1*. Therefore, despite inducing a mild knockdown (a 20% decrease in comparison to control), gRNA-3 was selected for further experiments. To determine the potential effect of circ_Satb1 downregulation on dendritic spines, DIV17 PHN cultures were used (Figure 5D). Green fluorescent protein (GFP) staining detected pCasRx_EGFP expression (transfected neurons) and dendritic spine density and morphology were assessed in dissociated neurons (Figure 5E and Supplementary Figure 5B). A significant decrease of branched-like dendritic spines was found upon circ_Satb1 knockdown, while spine number was unchanged (Figures 5F,G). These data indicate that reduced circ_Satb1 expression induces changes in dendritic spine morphology and maturation.

## Discussion

Different classes of ncRNAs are deregulated in epilepsy. For example, the current study and work by others show that circRNA levels are generally decreased at chronic stages of human and experimental mTLE (Gong et al., 2018; Lee et al., 2018; Li et al., 2018). Although circRNAs had previously been implicated in epilepsy, our work is the first to thoroughly characterize the expression profile and function of an epilepsy-associated circRNA, circ_Satb1. Our data reveal specific spatiotemporal and subcellular expression of circ_Satb1 in neurons and show a role for this circRNA in the regulation of dendritic spine morphology. This, together with the observed decrease of circ_Satb1 in mTLE and studies linking dendritic spine defects and epilepsy, support the hypothesis that altered circ_Satb1 levels may contribute to the synaptic pathology underlying epilepsy.

### Circ_Satb1 Expression in the Developing and Diseased Brain

Our data did not reveal marked differences in circRNA expression between brain samples from mTLE patients lacking (non-HS) or showing HS (+HS). This is somewhat unexpected given the strong expression of circRNAs in neurons and the neuronal loss that characterizes mTLE + HS (Blümcke et al., 2013). However, circ_Satb1 is not only enriched in mouse hippocampal neurons but is also detected in microglia and astrocytes (data not shown). Gliosis is a hallmark of mTLE + HS (Blümcke et al., 2013) and it is possible that enhanced glial circ_Satb1 expression compensates for reduced neuronal expression.

The mechanisms that regulate circ_Satb1 expression in mTLE or in general are currently unknown. By comparing circRNA, mRNA and protein expression from the *SATB1* locus, we observed deregulation of circ_Satb1 but not of *SATB1* mRNA or protein. Often circRNAs and mRNAs derived from the same locus display similar patterns of expression regulation. However, independent expression regulation has also been observed (Jeck et al., 2013; Salzman et al., 2013; Ashwal-Fluss et al., 2014; Rybak-Wolf et al., 2014; Xu et al., 2018). Our findings are in line with previous work showing no clear correlation between a set of mTLE-associated circRNAs and linear transcripts from the same loci (Gray et al., 2020). These observations suggest that changes in for example back-splicing rather than transcriptional activation of the *SATB1* locus may cause altered circ_Satb1 expression in the hippocampus of mTLE patients. This is interesting as altered splicing has been linked to epilepsy and seizures (Heinzen et al., 2007; Douaud et al., 2011; Baines et al., 2012; Lin et al., 2015; Cohen and Lambris, 2018; Thompson et al., 2020).

### CRISPR/CasRx-Mediated Knockdown of Circ_Satb1 Induces Dendritic Spine Defects

Knockdown of circRNA expression requires unique targeting of the back-splice junction to minimize effects on the linear transcript and can be challenging because of the presence of certain back-splice junction features (e.g., high G/C content, nucleotide repetitions). For circ_Satb1, the presence of a poly-T stretch at the back-splice junction restricted gRNA design possibilities. Using CRISPR/CasRx, a mild decrease in circ_Satb1 expression was achieved in neuronal cells. Further optimization (e.g., variable CasRx/gRNA ratios, knockdown or RNA collection at different DIVs, different transfection methods) did not lead to enhanced knockdown efficiency, nor did the use of other knockdown strategies (e.g., antisense LNA gapmeRs). Given the mild knockdown, it is plausible that the full impact of circ_Satb1 on dendritic spine morphology is much larger then could be shown in this study. Nevertheless, even small changes in circRNA expression can have significant impact. For example, *in vivo* depletion of *circHomer1a* (40% knockdown) in the orbitofrontal cortex induces marked cognitive defects in mice (Zimmerman et al., 2020).

Epilepsy is categorized as a synaptopathy and synaptic proteins are a main target of AEDs (Fukata and Fukata, 2017). Multiple studies using human tissue or animal models have reported changes in dendritic spine number and morphology in epilepsy (Swann et al., 2000; Wong, 2005; Wong and Guo, 2013). Here, we show that downregulation of circ_Satb1, as observed in human and experimental mTLE, induces a reduction in the number of complex spines. Dendritic spines are essential for synaptic plasticity and transmission and vary in shape, turnover, prevalence and synaptic activity (Berry and Nedivi, 2017). Mature spines, such as mushroom or branched spines, are prevalent in the adult brain and contain large excitatory synapses. In contrast, filopodial spines are more immature structures that are highly enriched during early postnatal development. Filopodial spines display high turnover and low synaptic function (Berry and Nedivi, 2017). Our observation that reduced circ_Satb1 triggers a decrease in the number of complex, mature spines is consistent with the presence of dysmorphic filopodia-like structures during epileptogenesis (Musto et al., 2016). Our analysis did not reveal defects in dendritic spine density. This could result from the relatively modest knockdown of circ_Satb1 but it is also possible that this circRNA has a specific function in controlling spine morphology and maturity. *In vitro* studies suggest a synaptogenesis model involving the transition from dendritic filopodia (lacking PSD-95) to mature spines (containing PSD-95) (Dailey and Smith, 1996; Marrs et al., 2001). Our data hint at the possibility that circ_Satb1 contributes to maturation and stabilization steps required for the formation of functional spines. Interestingly, transcripts co-expressed with circ_Satb1 in the human hippocampus have important roles in synaptic function (e.g., NRGN, CPLX1, SYNGR1, RPH3A, GABBR2, and KCNJ4) (S. Abul-Husn et al., 2009; Croning et al., 2009). A role for circ_Satb1 at synapses is also in line with its temporal expression profile during brain development. In contrast to our observations in mTLE tissue, mouse circ_Satb1 and *Satb1* display similar spatiotemporal patterns of expression during brain development and maturation. While circ_Satb1 expression in the mouse hippocampus initially decreases during early postnatal development, it increases from late postnatal to adult stages. At the cellular level this transition is marked by enriched synaptogenesis and changes in neurotransmitters and receptors (Semple et al., 2013). Other support for a synaptic role for circ_Satb1 derives from its enrichment in murine hippocampal synaptoneurosomes (Rybak-Wolf et al., 2014).

How circ_Satb1 influences dendritic spines or synapses in general is unknown. Described mechanism-of-actions for circRNAs include the competitive binding of miRNAs or RBPs (Kristensen et al., 2019). CircInteractome analysis of the circ_Satb1 sequence unveils several binding sites for RBPs, including for Fragile X mental retardation protein (FMRP) and human antigen R (HuR) (Dudekula et al., 2016). These RBPs are enriched at the synapse, where they regulate mRNA transport and translation, and have been implicated in brain disease (Sephton and Yu, 2015). While further studies are needed to establish the potential synaptic role of circ_Satb1, our data are the first to implicate a circRNA in the regulation of dendritic spine morphology.

### CircRNAs as Disease Biomarkers?

Correlation analysis showed a positive association between circ_Satb1 expression in mTLE patients and postoperative seizure-free period. This observation hints at the possibility that circ_Satb1 levels may have predictive value for surgery outcome. While this is an exciting hypothesis, further work is needed as the analysis is based on a small number of patients. Moreover, clinical information used in the correlation analysis did not take into account some confounding factors (e.g., presence of other comorbidities, AED usage, etc.) known to influence poor surgery outcome (Bell et al., 2017). Nevertheless, circRNAs have been identified as biomarkers for various diseases (Ma et al., 2020; Verduci et al., 2021) and it will be interesting to further explore their biomarker potential in the context of epilepsy. In this light it is also interesting to note that whereas circRNAs are generally downregulated at chronic stages of epilepsy (this study; Gong et al., 2018; Lee et al., 2018; Li et al., 2018), enhanced expression was found at specific stages of epileptogenesis (Gomes-Duarte et al., 2021). It is therefore possible that distinct circRNA levels or signatures mark different stages of the epilepsy disease process.

In conclusion, our study shows decreased expression of circRNAs in the hippocampus of human mTLE patients and identifies a role for one of these deregulated circRNAs, circ_Satb1, in regulating dendritic spine morphology. Given the reported dendritic spine and synaptic defects in human and experimental epilepsy, it will be interesting to explore the role of circRNAs in the control of synaptic function and plasticity in healthy and disease states in future studies.

## Data Availability Statement

The original contributions presented in the study are publicly available. This data can be found here: https://www.ncbi.nlm.nih.gov/geo, under the accession number GSE186334.

## Ethics Statement

All patients and control donors provided written informed consent for the use of their material and clinical information for research purposes (Vangoor et al., 2021). All procedures performed and the use of tissue and clinical information for research purposes were approved by the Institutional Review Board of University Medical Center Utrecht. Postmortem tissue was obtained from the Netherlands Brain Bank. The use of postmortem tissue for research purposes was approved by the Medical Ethics Board of the Amsterdam University Medical Center. All animal experiments were approved by local authorities in Utrecht (Animal Ethics Committee of Utrecht University) in compliance with Dutch law (Wet op de Dierproeven, 1996; revised 2014). All procedures were performed in accordance with EU regulations (Guideline 86/609/EEC; Directive 2010/63/EU).

## Author Contributions

AG-D contributed to methodology, investigation, formal analysis, visualization, and writing-original draft preparation. MTV contributed to investigation, analysis, and visualization. KS contributed to methodology, animal work, and investigation. MW, MB, JH, FRH, DR, MR-T, JK, IL, and NR contributed to methodology. PR, PE, and PG contributed to epilepsy surgery material. VRV contributed to methodology, investigation, formal analysis, visualization, and writing-review and editing. RJP contributed to conceptualization, resources, writing-review and editing, supervision, and funding acquisition. All authors contributed to the article and approved the submitted version.

## Conflict of Interest

MTV was employed by the company Omiics ApS. The remaining authors declare that the research was conducted in the absence of any commercial or financial relationships that could be construed as a potential conflict of interest.

## Publisher’s Note

All claims expressed in this article are solely those of the authors and do not necessarily represent those of their affiliated organizations, or those of the publisher, the editors and the reviewers. Any product that may be evaluated in this article, or claim that may be made by its manufacturer, is not guaranteed or endorsed by the publisher.

## Footnotes

1
https://pga.mgh.harvard.edu/primerbank/

2
http://cbl-gorilla.cs.technion.ac.il

3
www.biomath.info

## Supplementary Material

Supplementary Figure 1 RT-qPCR analysis of circRNAs in mTLE (age-colored). CircRNA expression (circ_Satb1, circ_Satb2, and circ_Lrp6) colored by the age of individuals, represented among postmortem controls and mTLE patients. Circ_Unc13c (hsa_circ_0103896) and circ_Syt1 (hsa_circ_0099287) were included as a control for expression changes, as predicted by RNA-seq. Color gradient indicates age (from younger, yellow to older, and blue). Dots represent box-plot R calculated outliers. No outliers were excluded during statistical analysis.

Supplementary Figure 2 Characterization of the ICK mice model of TLE. Nissl staining was used to assess neuron death at different time points in both contralateral (saline-injected) and ipsilateral (KA-injected) hippocampal sides. Note the prominent cell loss and dentate gyrus (DG) granule cell dispersion in the ipsilateral side at 30 days (d). Scale bar: 100 μm.

Supplementary Figure 3 Human and mouse circ_Satb1 isoforms. **(A)** Comparison of circ_Satb1 exonic sequences in *Homo sapiens* and *Mus musculus* using NCBI alignment tool “blastn.” **(B)** Back-splice junction sequencing of circ_Satb1 followed by custom DNA sequencing was used to confirm the back-splice junction sequence identity of circ_Satb1. Hippocampal material from P0 C57BL/6 pups was used as a cDNA template (bsj, back-splice junction; F, forward). **(C)** Detection of circ_Satb1 with single-molecule RNA *in situ* hybridization (smFISH) in primary hippocampal neurons. Neurons were stained with neuron-specific class III beta-tubulin (β-tubulin III) and nuclei with 4,6-diamidino-2-phenylindole (DAPI). Red arrows indicate circ_Satb1 signal. Scale bar: 25 μm.

Supplementary Figure 4 Circ_Satb1 co-expressed (mRNA) transcripts in hippocampal samples. Heatmap showing correlation strength between transcripts in human hippocampal samples. Pearson correlation coefficient was used (scale from blue = negative correlation, to red = positive correlation). Blue selection depicts region of interest.

Supplementary Figure 5 Experimental details of the dendritic spine analysis using CasRx-based knockdown. **(A)** Different gRNAs targeting the circ_Satb1 back-splice junction were characterized according to their composition and potential binding to the linear *Satb1* transcript. ***Potential binding to off-targets was investigated using tblastn (https://blast.ncbi.nlm.nih.gov/Blast.cgi) (F, forward; R, reverse; bp, base pairs; nt, nucleotides). **(B)** Dendrites at a distance ≥20 μm of the soma were considered (exclusion of soma-dendrites). Spine morphology analysis was performed along ≈ 100 μm dendritic length. Dendritic spines were classified as Branched, Mushroom, Stubby, Thin or Filopodia (1–5) according to visual detection using ImageJ plugin “Cell counter.”

Supplementary Table 1 DESeq2 analysis of circRNAs in mTLE versus control. List of all differentially expressed circRNAs detected after RNA-seq and DESeq2 analysis. CircRNAs are sorted by minimum *p*-value in mTLE (E) versus control tests (C/S). Cell colors represent the strength of significance (yellow = low, to red = high). *N* = 5 individuals were used per group (hg19, *Homo sapiens* genome assembly GRCh37; E, epilepsy; C/S, control; cx, cortex; hip, hippocampus; cyt, cytoplasm; nuc, nucleus; w0, mTLE non-HS patients; w4, mTLE + HS patients; padj, adjusted *p*-value).

Supplementary Table 2 List of oligonucleotides used in this study. Primers were designed using Primer-BLAST online software (https://www.ncbi.nlm.nih.gov/tools/primer-blast/) or obtained from literature (mouse circ_Homer1) (You et al., 2015), and purchased from IDT-DNA. The following criteria were met during primer design: 18–30 bp length, 50–65°C melting temperature (Tm), 40–65% %G/C content, minimum of self-complementary regions and amplicon size ≤250 bp.

Supplementary Table 3 NormFinder analysis to identify genes for normalization. NormFinder (*.xla, MS Excel 2003 v0.953) was used to determine the most stable normalization gene(s) to be used in RT-qPCR assays aiming to assess expression changes between controls and mTLE patients (human) or saline- and KA-injected animals (mouse). The most optimal gene, or combination of genes, is determined by their stability value: the lower this value, the more stable the gene or gene combination. Transformed Ct values were used in the analysis. tbb-2 from *Caenorhabditis elegans* (c.e.) was used as a spike-in to control for possible artifacts during cDNA synthesis (E1-E7, human mTLE; C1-C7, controls; KA1-KA33, KA-injected mice; Sal4-36, saline-injected mice; tbb-2, tubulin beta-2 chain; ICK, intracortical kainate).

Supplementary Table 4 List of antibodies used in this study. Information on primary **(A)** and secondary **(B)** antibodies used in this study.

Supplementary Table 5 Clinical information of mTLE patients used in RT-qPCR studies. Seizure frequency/duration information was grouped into classes (I-IX) to facilitate downstream analysis. No discrimination was made for epileptic episodes and all types were considered (complex; loss of consciousness; complete or partial tonic seizures; epileptic episodes [auras]). Recurrence was evaluated over a period of 5 years post-resection surgery. Clinical information was correlated with circ_Satb1/2 expression in mTLE (RT-qPCR). When no info was available for a specific patient this was excluded from the analysis.

Supplementary Table 6 Gene expression correlation values of circ_Satb1 and mRNA transcripts in the hippocampus. Correlation analysis was used to show co-expression patterns between circ_Satb1 expression changes and defined groups of transcripts deregulated in the hippocampus. Correlation values (r) are represented in each cell. Both nuclear and cytoplasmic hippocampi samples were used in the analysis.

Supplementary Table 7 GO analysis of mRNA transcripts co-expressed with circ_Satb1 in the hippocampus. GO analysis was performed using the GOrilla interface. The background list included all correlated transcripts (genes) whereas the target list included all transcripts (genes) found to be most strongly co-expressed with circ_Satb1 and among each other. *P*-value < 0.01 was set as a threshold for the GOrilla analysis. Enrichment was calculated as −log_2_P-value and used in the graphical representation (GO, gene ontology; FDR, false discovery rate).

## Abbreviations

AEDs: anti-epileptic drugs
c.e.: C. elegans
CA1–4: Cornu Ammonis 1–4
cDNA: complementary DNA
circRNA: circular RNA
Ct: cycle threshold
d: days
DAPI: 4,6-diamidino-2-phenylindole
DE: differentially expressed
DG: Dentate gyrus
DIV: days *in vitro*
FC: fold-change
FDR: False discovery rate
FMRP: Fragile X mental retardation protein
*Gapdh*: Glyceraldehyde-3-Phosphate Dehydrogenase
GO: gene ontology
hg19: human genome
*Hprt1*: Hypoxanthine Phosphoribosyltransferase 1
HS: hippocampal sclerosis
HuR: human antigen R
ICK: intracortical kainate
ILAE: International League Against Epilepsy
KA: kainic acid
LRP6: LDL Receptor Related Protein 6
miRNA: microRNA
mRNA: messenger RNA
mTLE: mesial Temporal Lobe Epilepsy
ncRNAs: non-coding RNAs
NeuN: neuronal nuclei
Neuro-2a: mouse neuroblastoma cell line
ns: non-specific
padj: adjusted *p*-value
PCR: polymerase chain reaction
PHN: primary hippocampal neurons
RBPs: RNA-binding proteins
RNA-seq: RNA-sequencing
RNase R: ribonuclease R
*RPII*: RNA-polymerase II
rRNA: ribosomal RNA
RT: room temperature
RT-qPCR: reverse transcriptase quantitative PCR
SATB1: special AT-rich sequence binding protein 1
SATB2: Special AT-rich sequence-binding protein 2
SD: standard deviation
SE: *status epilepticus*
SEM: standard error of the mean
s.c.: subcutaneous
*TBP*: TATA-Box Binding Protein
TLE: Temporal Lobe Epilepsy
β-actin: Beta-actin
β-Tubulin III: Neuron-specific class III beta-tubulin.
