# Supplementary figures and images for: Expression of Circ_Satb1 Is Decreased in Mesial Temporal Lobe Epilepsy and Regulates Dendritic Spine Morphology
Source: Front Mol Neurosci. 2022 Mar 3;15:832133. doi: 10.3389/fnmol.2022.832133 (PMC8927295; doi:10.3389/fnmol.2022.832133)

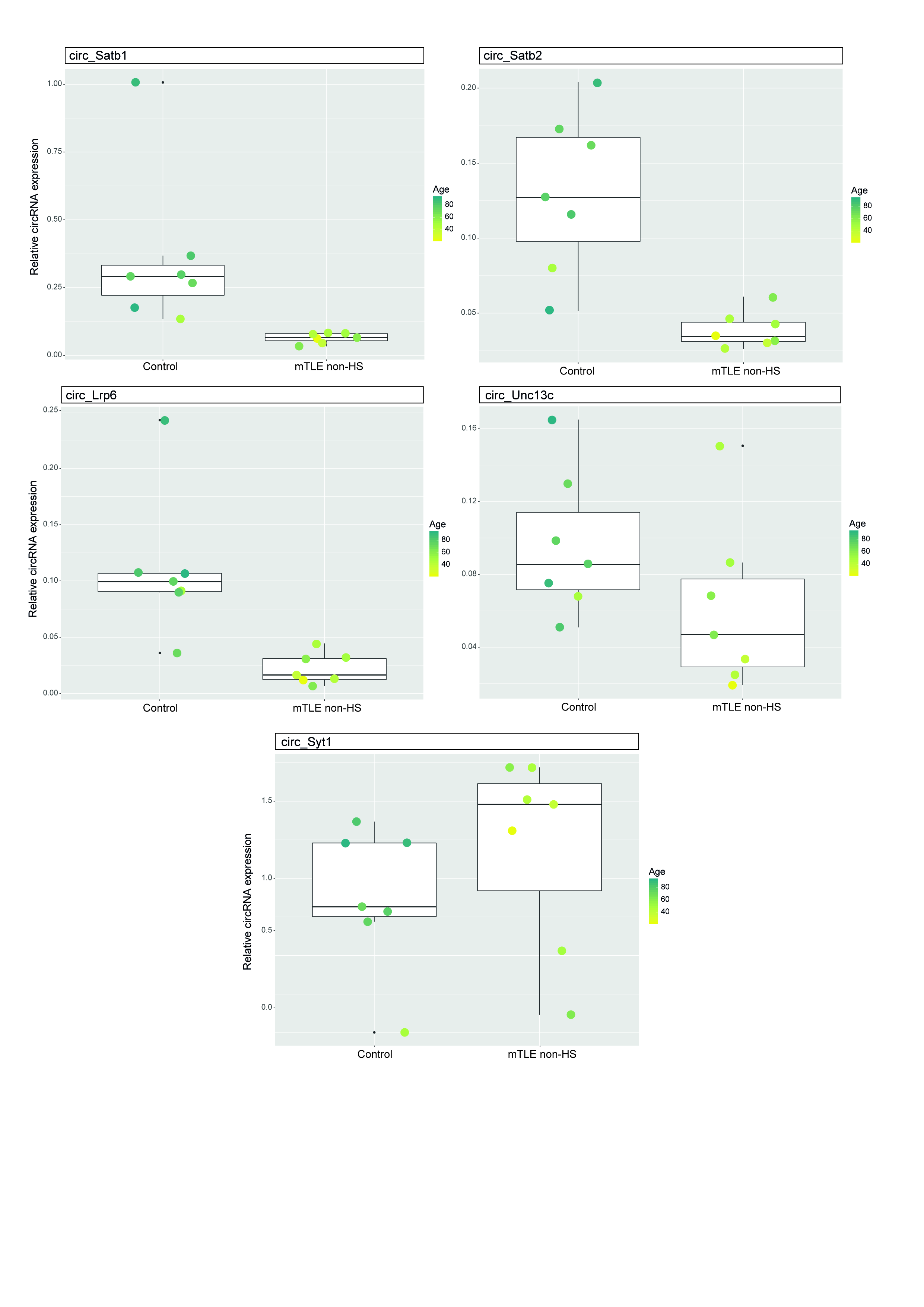

Supplement: Supplementary Figure 1 — RT-qPCR analysis of circRNAs in mTLE (age-colored). CircRNA expression (circ_Satb1, circ_Satb2, and circ_Lrp6) colored by the age of individuals, represented among postmortem controls and mTLE patients. Circ_Unc13c (hsa_circ_0103896) and circ_Syt1 (hsa_circ_0099287) were included as a control for expression changes, as predicted by RNA-seq. Color gradient indicates age (from younger, yellow to older, and blue). Dots represent box-plot R calculated outliers. No outliers were excluded during statistical analysis. [file Image_1.TIF]

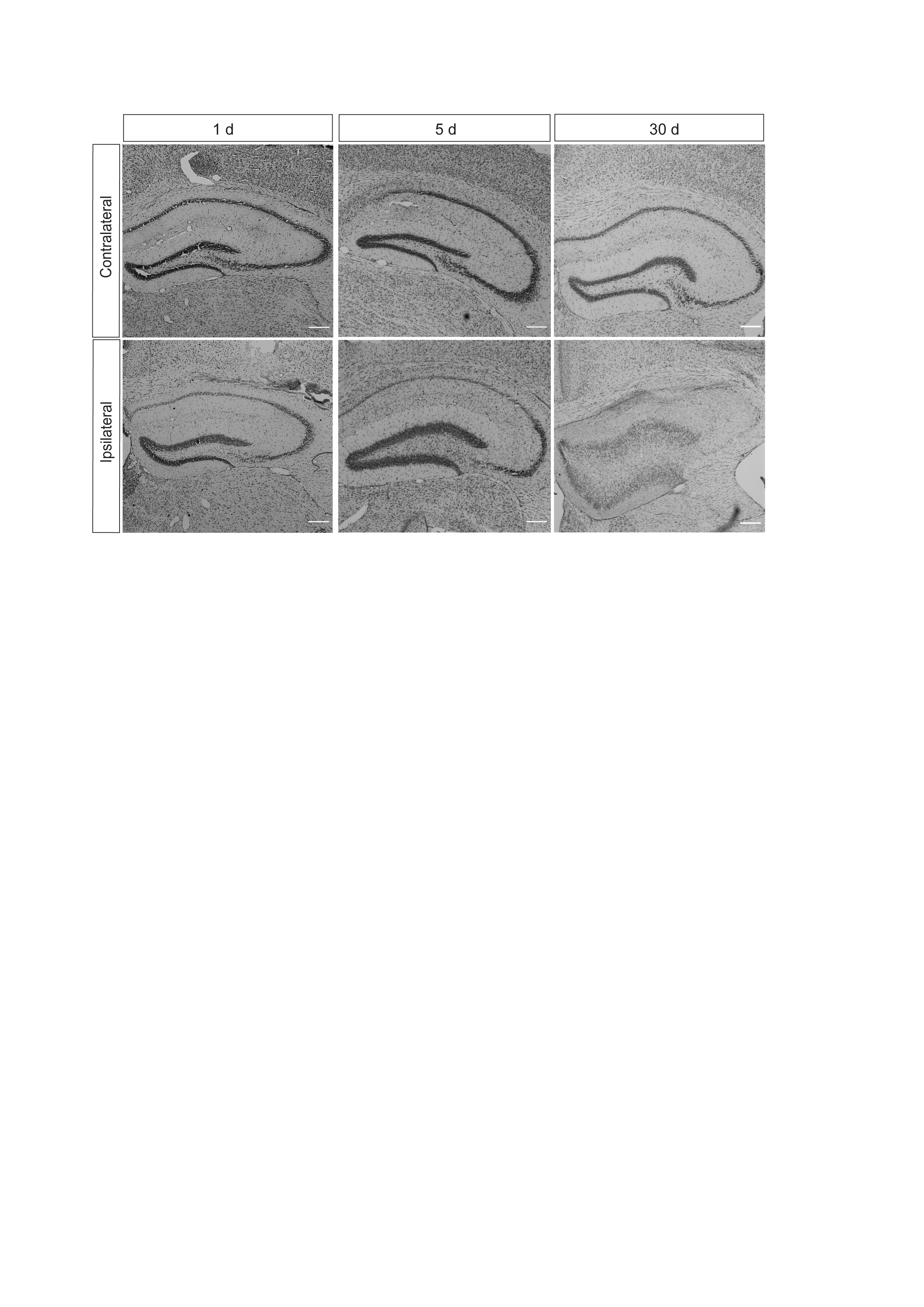

Supplement: Supplementary Figure 2 — Characterization of the ICK mice model of TLE. Nissl staining was used to assess neuron death at different time points in both contralateral (saline-injected) and ipsilateral (KA-injected) hippocampal sides. Note the prominent cell loss and dentate gyrus (DG) granule cell dispersion in the ipsilateral side at 30 days (d). Scale bar: 100 μm. [file Image_2.TIF]

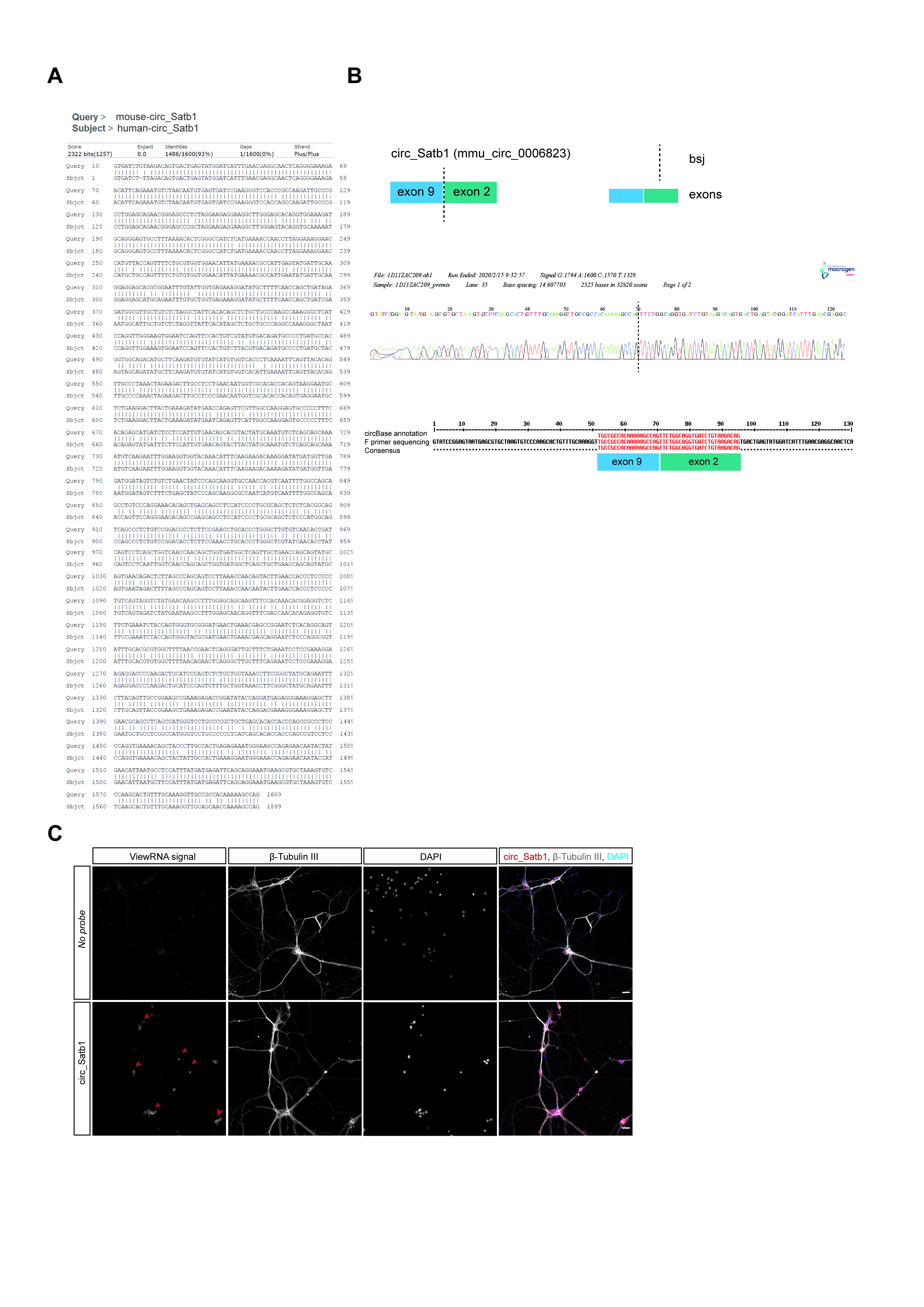

Supplement: Supplementary Figure 3 — Human and mouse circ_Satb1 isoforms. (A) Comparison of circ_Satb1 exonic sequences in Homo sapiens and Mus musculus using NCBI alignment tool “blastn.” (B) Back-splice junction sequencing of circ_Satb1 followed by custom DNA sequencing was used to confirm the back-splice junction sequence identity of circ_Satb1. Hippocampal material from P0 C57BL/6 pups was used as a cDNA template (bsj, back-splice junction; F, forward). (C) Detection of circ_Satb1 with single-molecule RNA in situ hybridization (smFISH) in primary hippocampal neurons. Neurons were stained with neuron-specific class III beta-tubulin (β-tubulin III) and nuclei with 4,6-diamidino-2-phenylindole (DAPI). Red arrows indicate circ_Satb1 signal. Scale bar: 25 μm. [file Image_3.TIF]

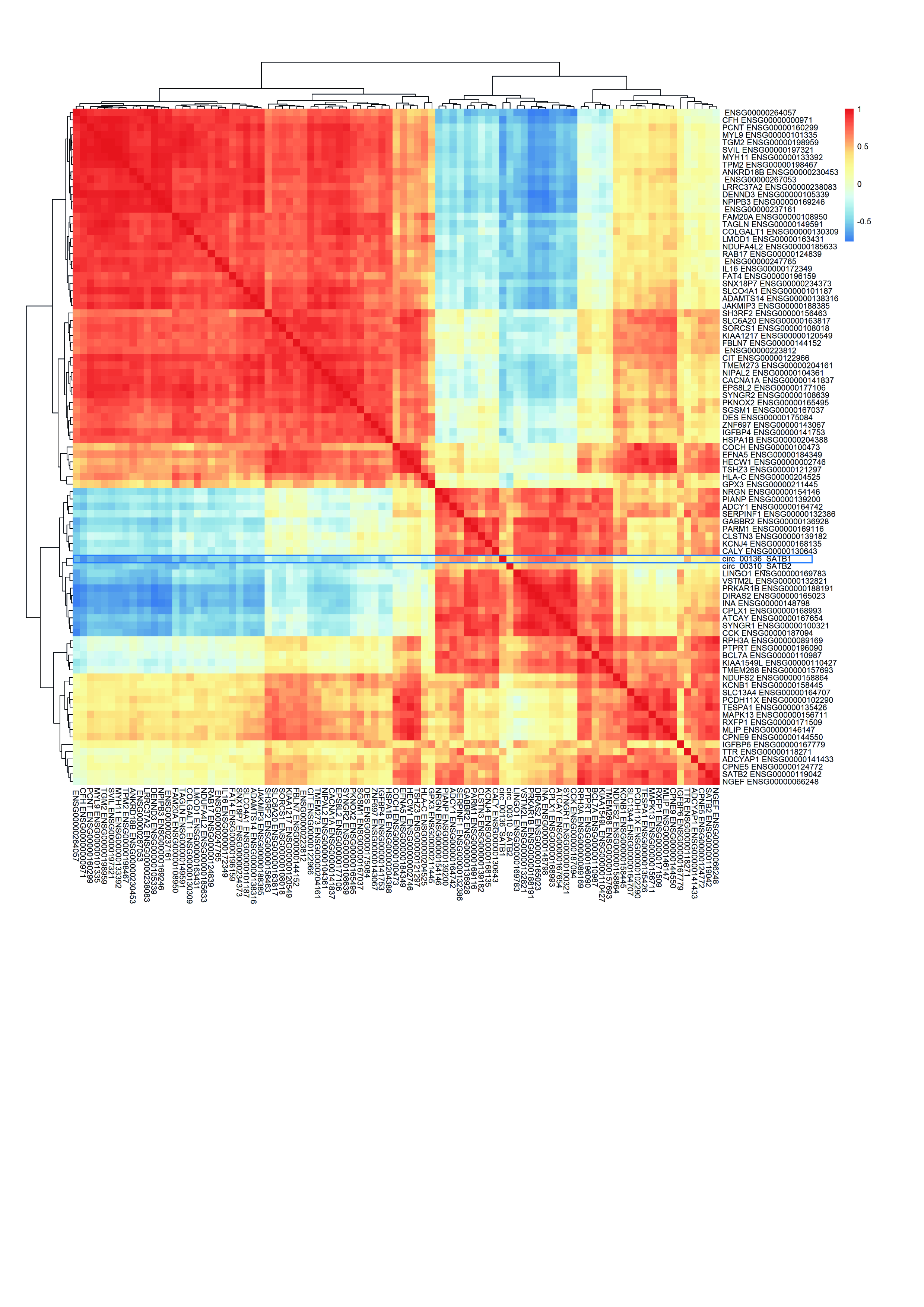

Supplement: Supplementary Figure 4 — Circ_Satb1 co-expressed (mRNA) transcripts in hippocampal samples. Heatmap showing correlation strength between transcripts in human hippocampal samples. Pearson correlation coefficient was used (scale from blue = negative correlation, to red = positive correlation). Blue selection depicts region of interest. [file Image_4.TIF]

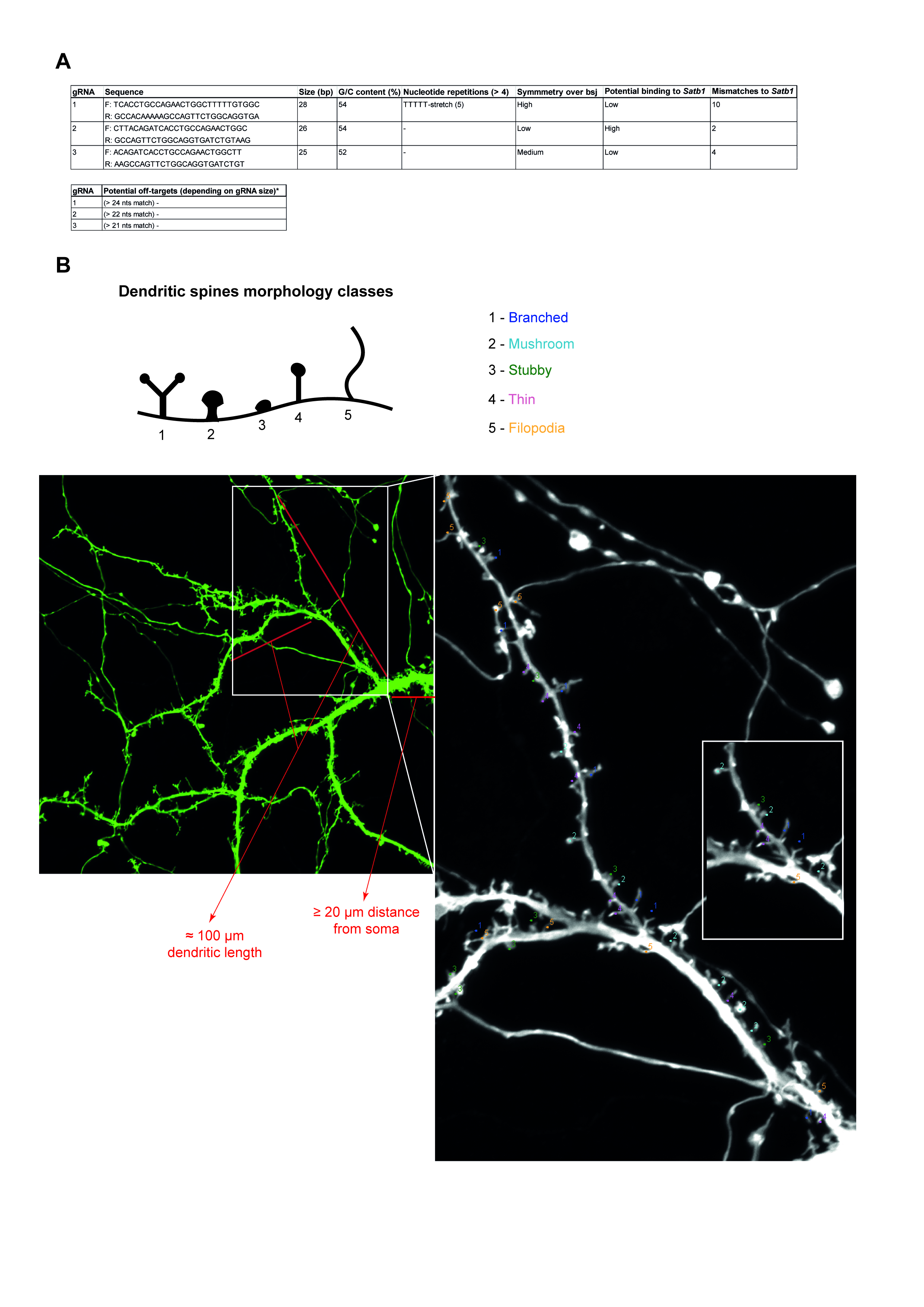

Supplement: Supplementary Figure 5 — Experimental details of the dendritic spine analysis using CasRx-based knockdown. (A) Different gRNAs targeting the circ_Satb1 back-splice junction were characterized according to their composition and potential binding to the linear Satb1 transcript. *Potential binding to off-targets was investigated using tblastn (https://blast.ncbi.nlm.nih.gov/Blast.cgi) (F, forward; R, reverse; bp, base pairs; nt, nucleotides). (B) Dendrites at a distance ≥20 μm of the soma were considered (exclusion of soma-dendrites). Spine morphology analysis was performed along ≈ 100 μm dendritic length. Dendritic spines were classified as Branched, Mushroom, Stubby, Thin or Filopodia (1–5) according to visual detection using ImageJ plugin “Cell counter.” [file Image_5.TIF]
